# Cholangiocarcinoma cells direct fatty acids to support membrane synthesis and modulate macrophage phenotype

**DOI:** 10.1097/HC9.0000000000000717

**Published:** 2025-05-23

**Authors:** Michele Dei Cas, Stefania Mantovani, Barbara Oliviero, Aida Zulueta, Linda Montavoci, Monica Falleni, Delfina Tosi, Camillo Morano, Sara Penati, Annalisa Chiocchetti, Riccardo Sinella, Camilla Barbero Mazzucca, Matteo Donadon, Cristiana Soldani, Gaetano Piccolo, Matteo Barabino, Paolo Pietro Bianchi, Marcello Maestri, Ana Lleo, Jesus M. Banales, Mario U. Mondelli, Anna Caretti

**Affiliations:** 1 Department of Health Sciences, University of Milan, Milan, Italy; 2 Research Department, Division of Clinical Immunology—Infectious Diseases, Fondazione IRCCS Policlinico San Matteo, Pavia, Italy; 3 Istituti Clinici Scientifici Maugeri IRCCS, Neurorehabilitation Unit of Milan Institute, Milan, Italy; 4 Health Sciences Department, Pathology Division, University of Milan, Milan, Italy; 5 Department of Pharmaceutical Sciences, University of Milan, Milan, Italy; 6 Department of Health Sciences, University of Piemonte Orientale, Novara, Italy; 7 Department of Surgery, University Maggiore Hospital della Carità, Novara, Italy; 8 Laboratory of Hepatobiliary Immunopathology, IRCCS Humanitas Research Hospital, Rozzano, Milan; 9 Department of Health Sciences, General Surgery Unit, University of Milan, Milan, Italy; 10 Division of General Surgery 1, Fondazione IRCCS Policlinico San Matteo, Pavia, Italy; 11 Department of Biomedical Sciences, Humanitas University, Milan, Italy; 12 Department of Gastroenterology, Division of Internal Medicine and Hepatology, IRCCS Humanitas Research Hospital, Rozzano, Milan, Italy; 13 Department of Liver and Gastrointestinal Diseases, Biogipuzkoa Health Research Institute-Donostia University Hospital, University of the Basque Country (UPV/EHU), Ikerbasque, San Sebastian, Spain; 14 Department of Biochemistry and Genetics, School of Sciences, University of Navarra, Pamplona, Spain; 15 Department of Internal Medicine and Therapeutics, University of Pavia, Pavia, Italy

**Keywords:** cholangiocarcinoma, fatty acid, lipid droplets, liver cancer, macrophages

## Abstract

**Background and Aims:**

Cholangiocarcinoma (CCA) is a globally rare, increasingly incident cancer. Metabolic reprogramming is common in cancer cells, and altered lipid homeostasis favors tumor development and progression. Previous studies have described lipid deregulation in HCC cells, while in CCA, the lipidome profile is still poorly characterized.

**Methods:**

We used liquid chromatography–tandem mass spectrometry to examine the lipid level profile of intrahepatic CCA (iCCA) and non-tumor surrounding tissue from patients, as well as in patients’ and healthy controls’ sera.

**Results:**

All lipid classes were upregulated in tumor specimens and iCCA-derived sera. Newly synthesized fatty acids (FAs) accumulated in iCCA and were only marginally directed to mitochondrial β-oxidation and scarcely folded in lipid droplets as neutral species. Metabolic flux assay showed that FAs were instead redirected toward plasma membrane formation and remodeling, being incorporated into phospholipids and sphingomyelin. A distinct lipid droplet and macrophage distribution was revealed by immunohistochemistry and Imaging Mass Cytometry. Lipid droplets were fewer in iCCA than in normal tissue and present mainly in the intratumoral fibrous septa and in M2 macrophages. Monocytes modified their lipid content and phenotype in the presence of iCCA cells, and the same effect could be recapitulated by FA supplementation.

**Conclusions:**

Our results reveal a profound alteration in the lipid content of iCCA tissues and demonstrate that FA accumulation prompts iCCA aggressiveness by supporting membrane biogenesis, generating bioactive lipids that boost proliferation, and by modifying macrophage phenotype.

## Graphical abstract

**Figure:**
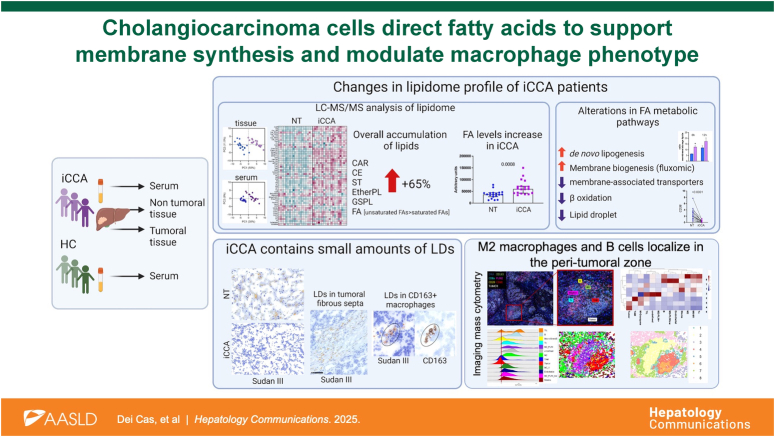

## INTRODUCTION

Intrahepatic cholangiocarcinoma (iCCA) belongs to a heterogeneous group of malignancies arising along the biliary tree. iCCA represents the second most common primary liver malignancy after HCC. Due to a lack of early symptoms and reliable diagnostic markers, patients frequently present at an advanced stage, which results in very limited treatment options. When feasible, liver resection is the treatment of choice, followed by locoregional therapy and systemic chemotherapy, according to recently released EASL-ILCA clinical practice guidelines.^1^ Liver transplant represents a treatment option under investigation protocols in patients with very early iCCA, in major referral centers.^2^

Metabolic reprogramming is a hallmark of cancer to sustain tumor growth and anabolic pathways. Indeed, cancer cells strictly depend on glucose for energy fueling, and enhanced glycolysis generates glucose-derived carbons that are converted into nucleotides, proteins, and lipids.^3^ Lipids are crucial for cell survival, being components of biological membranes, energy suppliers via fatty acid (FA) oxidation, and bioactive mediators. Altered lipid homeostasis contributes to the rapid progression of different cancers, and inhibition of lipid biosynthesis has been targeted to reduce tumor growth.^4^

Several studies have described lipidomic deregulation in HCC, while in CCA, a lipidome profile is still lacking.^5^ A previous metabolomic study conducted in serum from iCCA patients and control subjects identified different profiles.^6^ While the PCA model showed no separation in the serum metabolite profile between iCCA and control subjects, a total of 52 metabolites were altered in sera from patients, where phosphatidylcholines (PCs), amino acids, sphingomyelins (SMs), and sterols (STs) were the most abundant metabolite families found to be altered. FA dysregulation has been widely associated with tumor progression. FA synthesis is downregulated in iCCA while FA uptake-related proteins are enhanced. In vitro and in a mouse model of AKT/NICD-driven cholangiocarcinogenesis, FA synthase (FASN) and de novo lipogenesis were not required (reviewed in Raggi et al^7^). In contrast, another study associated high FASN expression with advanced-stage CCA and a shorter patient survival. Furthermore, FASN knockdown inhibited growth, migration, invasion, and cell cycle progression, and induced apoptosis in CCA cells.^8^ The conflicting data on the mechanism of FA accumulation might result from the different availability of FA in the extracellular compartment.^7^ FAs are precursors of triglycerides (TG), phospholipids (PL), and bioactive mediators such as sphingolipids (SPL), which are involved in processes relevant to CCA. Among SPL, sphingosine-1-phosphate (S1P) derived from ceramide (Cer) via phosphorylation of sphingosine kinase 1 (SPHK1) promotes proliferation and survival of iCCA, augmenting its aggressiveness.^9^ Cholesterol (Chol) and derived bile acids (BAs) are elevated in CCA and may also contribute to CCA development.^10^ The tumorigenic potential of different CCA cell lines correlates with FA and lipoprotein incorporation into TG storage. Indeed, CCA cell lines with greater proliferative potential rely on upregulated FA oxidation to meet energy demand.^11^ Though FAs are essential for cancer growth, excessive cellular accumulation is harmful to cells because of the generation of damaging bioactive lipids. Indeed, cells avoid lipotoxicity by accelerating mitochondrial FA β-oxidation^12^,^13^ and by safely storing FA into lipid droplets (LDs) as neutral lipids (TG and Chol esters).^14^ LDs are cytoplasmic vesicles bounded by a monolayer of PLs with a hydrophobic core of neutral lipids. Several studies have suggested that LDs are associated with cancer cell proliferation, resistance to death and aggressiveness,^14^ and support cancer stem cells functionality.^15^ Interestingly, there is evidence of LD involvement in tumor escape from immune surveillance. Of note, LD regulates the immune suppressive phenotype of tumor-associated macrophages (TAM), the main tumor-infiltrating leukocyte subset that promotes malignant progression^16^,^17^ and impairs dendritic cell ability to effectively stimulate antigen-specific T cells.^18^

In this study, we investigated the iCCA lipidome profile by liquid chromatography–tandem mass spectrometry (LC–MS/MS) analysis of iCCA tissue and matched non-tumor (NT) surrounding tissues from biopsies obtained from iCCA patients. Sera from iCCA patients and healthy subjects (HC) were also analyzed. Patients’ serum and tumor specimens showed an overall increase of lipid classes compared with non-tumor tissue and healthy controls’ serum. FA accumulated in iCCA were hardly directed to mitochondrial β-oxidation or built up in LD as energy-reservoir TG, but were directed to membrane formation and remodeling. Indeed, tumor tissue displayed only few LDs, which were mainly localized in the fibrous septa and accumulated within M2 macrophages. Moreover, tumor cells modulate the lipid content and phenotype of monocytes toward M2, and the same effect could be recapitulated by supplementing monocytes with FA.

## METHODS

### Study subjects

Paired peripheral blood and surgically resected iCCA specimens, along with matched, NT tissue from a non-adjacent liver site, were obtained from treatment-naïve patients recruited at Fondazione IRCCS Policlinico San Matteo, Pavia; IRCCS Humanitas Research Hospital; and ASST Santi Paolo e Carlo Hospital, Milan, Italy. Tissue samples were stored in tissue storage solution (Miltenyi Biotec, Bergisch Gladbach, Germany) or RNA later (Merck, Darmstadt, Germany). Serum derived from iCCA patients and healthy controls (HCs; 12 females and 11 males, median age 65.4 years) was obtained from whole blood after clotting by centrifugation. The study was conducted in accordance with the Declaration of Helsinki (2013) and the Declaration of Istanbul (2018), and approved by the Ethics Committee of Fondazione IRCCS Policlinico San Matteo, Pavia (protocol numbers: P-20140031379 and P-20190104922). Patients’ characteristics are listed in Supplemental Table S1 and Supplemental Figure S12, http://links.lww.com/HC9/B984.

### RNA extraction, qPCR, and ddPCR

Tissue RNA extraction was performed using a TRIzol reagent (Thermo Fisher Scientific) with a gentleMACS Dissociator (Miltenyi Biotec). RNA was further treated with DNase on column using the RNeasy Plus Mini kit (Qiagen), according to the manufacturer’s instructions. First-strand cDNA was synthesized from 1 µg of total RNA with the High-Capacity cDNA Reverse Transcription Kit, following the manufacturer’s instructions (Thermo Fisher Scientific). The SsoAdvanced Universal SYBR Green Supermix (Bio-Rad) was used, and qPCR data were analyzed using the 2^−∆Ct^ method. Gene expression was normalized to hypoxanthine phosphoribosyltransferase 1 (HPRT1) expression. The primers are listed in Supplemental Table S2, http://links.lww.com/HC9/B984. Digital droplet PCR (ddPCR) was performed with the ddPCR Supermix for probes with the following primers: TATA-Box Binding Protein (TBP) dHsaCPE5058363 and FBP1 dHsaCPE5039776 (Bio-Rad). The total mix was placed into the 8-channel cartridge, and the droplets were generated with the QX200 droplet generator (Bio-Rad). Droplets were transferred to an Eppendorf 96-well plate (Eppendorf) and placed into the T100 Thermal Cycler (Bio-Rad) according to the manufacturer’s instructions. Droplets were subsequently read automatically by the QX200 droplet reader (Bio-Rad), and the data were analyzed with the QuantaSoft analysis PRO version 1 software (Bio-Rad).

Complete technical details are reported in the Supplemental Information and in the Supplemental Tables S3-S5, http://links.lww.com/HC9/B984. Tissues were prepared for LC–MS/MS and LC–MS for lipid profile characterization, as well as for Western blotting to analyze protein levels. Quantitative analysis of FA-labeled lipids, total lipids, and SPL was described in the Supplemental Information, http://links.lww.com/HC9/B984. Primary iCCA and NHC cell cultures and metabolic flux analysis, co-cultures, and palmitic acid treatment were established as described in the Supplementary Information and﻿ in the Supplemental Table S6, http://links.lww.com/HC9/B984. Tissue sections from patients prepared for histochemistry, immunohistochemistry, Imaging Mass Cytometry staining, acquisition, and data analysis were detailed in the Supplementary Information, http://links.lww.com/HC9/B984. Statistical analysis was performed using GraphPad Prism 10. We used parametric and non-parametric Wilcoxon or Mann–Whitney tests to compare paired or unpaired data, respectively, as detailed in the legend. A *p* value ≤0.05 was deemed statistically significant. Data from untargeted lipidomics were analyzed using MetaboAnalyst 4.0 for data processing, normalization (log-transformed and auto-scaled), and multivariate analysis such as Partial Least Squares Discriminant Analysis (PLSDA).

## RESULTS

### Fatty acids accumulate in iCCA-derived tissue and sera

**FIGURE 1:**
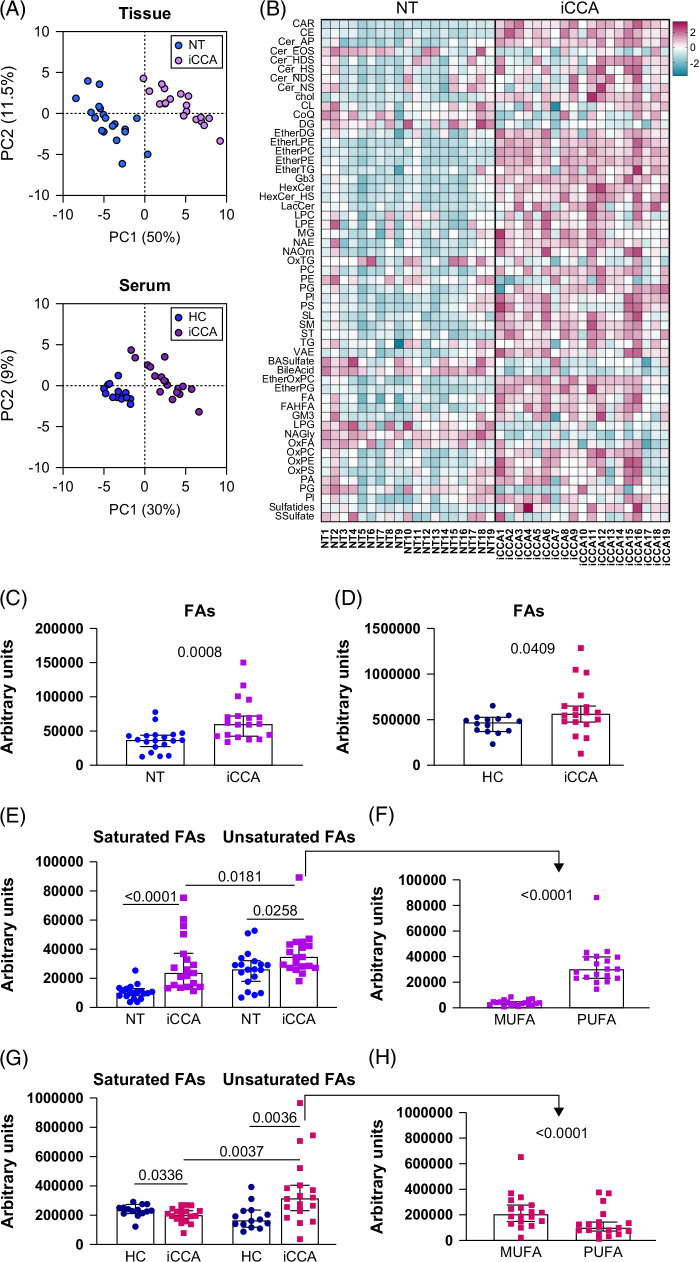
Lipidomic profile was altered in iCCA-derived tissue and sera. (A) Two-dimensional representation of PCA of the untargeted lipidomic profile of NT (light blue) and matched iCCA (pink) tissues (n=19, upper panel), and in HC-derived (light blue) and iCCA-derived (pink) sera (HC=14; iCCA=18, lower panel). (B) Heatmap showing the relative concentration of lipid subclasses in NT and matched iCCA tissues (n=19), using normalized intensities of lipids reordered in classes. (C) Analysis of FAs in NT and matched iCCA tissues (n=19). (D) Analysis of FAs in HC and iCCA-derived sera (HC=14; iCCA=18). (E) Analysis of saturated and unsaturated FAs in NT and matched iCCA tissues (n=19). iCCA-derived unsaturated FAs were separated into MUFA and PUFA (F). (G) Analysis of saturated and unsaturated FAs in HC-derived and iCCA-derived sera (HC=14; iCCA=18). iCCA-derived unsaturated FAs were separated into MUFA and PUFA (H). All lipidomic profiles were performed using high-resolution mass spectrometry. Shown are medians and 95% CI. The Wilcoxon test was used to compare NT and matched iCCA tissue, whereas Mann–Whitney was applied in comparing data from HC-derived and iCCA-derived sera. Abbreviations: FAs, fatty acids; HC, healthy control; iCCA, intrahepatic cholangiocarcinoma; MUFA, monounsaturated fatty acid; NT, non-tumoral; PUFA, polyunsaturated fatty acid.

We determined the untargeted lipidomic profile of human iCCA tissue, paired NT tissues, and sera derived from iCCA patients and HC by high-resolution mass spectrometry. There was a clear-cut separation between iCCA and NT tissues (principal component 1, PC1 50%) as well as between iCCA-derived sera and HC (PC1 30%), as shown by principal component analysis (PCA), indicating lipid rewiring in iCCA tissue and serum (Figure 1A). The spatial regions occupied by the 2 different tissues—iCCA and NT—in the PCA showed no overlap in either lipid profile derived from tissues or sera (Figure 1A). In tumor tissue, the overall accumulation of lipids was evident with a mean of +65%. The lipid pattern of tumor tissues was quantitatively different, among which acylcarnitine (CAR, fold-change, FC 33.0), cholesteryl esters (CE, FC 61.8), ST (FC 10.7), ether-phospholipids (EtherPL, FC 9.62), and glycosphingolipids (GSPL, FC 5.95) were upregulated whereas BA and their sulfate (FC 0.1 and 0.49, respectively), N-acylglycines (NAGly, FC 0.06) were downregulated, as illustrated in the heatmap (Figure 1B). The data indicated that most lipid classes were enhanced in iCCA and only a minority of them (12/56) were diminished. When we stratified patients according to metabolic dysfunction–associated fatty liver disease (MASLD) comorbidity, the PCA analysis of the lipid profile of tumor tissue and serum showed no separation between MASLD-associated iCCA and other patients (Supplemental Figure S1, http://links.lww.com/HC9/B984). Analysis of 55 classes of lipid showed no difference in MASLD-associated iCCA tissue compared with other patients (Supplemental Figure S2, http://links.lww.com/HC9/B984).

LC–MS/MS analysis revealed that FAs were predominantly enriched in iCCA tissue compared with NT tissue (Figure 1C), as well as in serum from iCCA patients compared with HC (Figure 1D). There was an enrichment in saturated and unsaturated FA in iCCA tissue, the latter being prevalent (Figure 1E). Among iCCA-derived unsaturated FA, polyunsaturated FA (PUFA) were 8-fold higher than monounsaturated FA (MUFA) (Figure 1F). Of note, unsaturated FAs were also increased in iCCA patients’ sera (Figure 1G), though in this case, MUFA were significantly higher than PUFA (Figure 1H). The overall content of PUFA was higher in iCCA compared with NT tissue, while MUFA was equally distributed (Supplemental Figures S3A, B, http://links.lww.com/HC9/B984). There was an increase in both unsaturated FA classes in iCCA patients’ sera compared with HC (Supplemental Figure S3D, http://links.lww.com/HC9/B984). An over 2-fold increase of fatty acyl esters of hydroxy FA (FAHFA) was also observed in iCCA biopsies (Supplemental Figure S3E, http://links.lww.com/HC9/B984).

**FIGURE 2:**
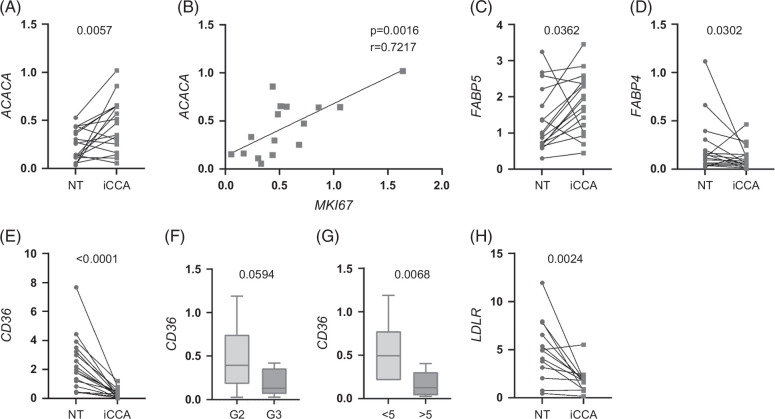
Fatty acid synthesis is increased in iCCA. (A) Expression of the *ACACA* gene was measured in NT and matched iCCA tissues (n=17). A paired *t* test was used to compare data. (B) The correlation between *ACACA* and *MKI67* expression was analyzed in 16 iCCA tumor tissues. (C–E and H) Expression of the membrane-associated transporters *FABP5*, *FABP4*, *CD36*, and *LDLR* gene in NT and matched iCCA tissues (*FABP5*, n=17; *FABP4*, n=16; *CD36*, n=17; *LDLR*, n=13). Wilcoxon test was used to compare data, except for *FABP5* expression, where a paired *t* test was used. (F and G) *CD36* expression in G2 (n=8) and G3 (n=9) tumors and in iCCA tissue stratified according to tumor size in millimeters (iCCA <5, n=7; iCCA >5, n=9). Median, minimum, and maximum values are shown in the box and whiskers plots; the unpaired *t* test was used to compare data. Abbreviations: ACACA, acetyl-CoA carboxylase alpha; CD36, cluster of differentiation 36; iCCA, intrahepatic cholangiocarcinoma; LDLR, low-density lipoprotein receptor; NT, non-tumoral.

To assess whether FA were elevated as a result of higher synthesis, we determined the expression of acetyl-CoA carboxylase alpha (*ACACA*) that catalyzes the rate-limiting step of de novo lipogenesis. *ACACA* transcription was higher in iCCA tissue than in NT tissue (Figure 2A) and positively correlated with the proliferation marker Ki-67 (*MKI67*) (Figure 2B). When iCCA samples were stratified according to tumor grade, the *ACACA* transcript was found to be upregulated in moderately differentiated (G2) tumors compared with NT tissues (Supplemental Figure S4A, http://links.lww.com/HC9/B984). Besides being newly synthesized, FA could be actively imported into the cells by several membrane-associated transporters such as FA translocase/cluster of differentiation 36 (FAT/CD36), FA binding proteins (FABPs), and FA transport proteins (FATPs). *FABP5* expression was higher in iCCA tissue (Figure 2C) while the expression of *FABP4* and *CD36* was downregulated in iCCA tissue (Figures 2D, E) regardless of differentiation (Supplemental Figure 4B, http://links.lww.com/HC9/B984), although there was a trend toward downregulation of *CD36* transcription in G3 compared with G2 tumors (Figure 2F). Larger tumors showed a strong reduction in the expression of *CD36* (Figure 2G). Also, the expression of the low-density lipoprotein receptor *(LDLR)*, which uptakes low-density lipoprotein/cholesterol, was downregulated (Figure 2H).

Emerging evidence showed that high macropinocytic activity is a hallmark of many human tumors, which use this adaptation to scavenge extracellular nutrients for fueling cell growth.^19^ Macropinocytosis is induced by hypoxia in different HCC cell lines^20^ harnessing extracellular protein as a nutrient to survive. FAs were also actively taken up in cancer cells via macropinocytosis.^21^ In pancreatic cancer cells, lipid synthesis potentiates expression of macropinocytosis-associated genes, including *SDC1*, *DNM2*, via the *ZIP4* (solute carrier family 39 member 4) pathway.^22^ Here we determined the expression of *ZIP4*, *DNM2*, and *SDC1* in iCCA tissue. *ZIP4* and *DNM2* transcription was higher in iCCA tissue than in NT tissue (Supplemental Figures S5A, B, http://links.lww.com/HC9/B984) while *SDC1* level was lower in iCCA tissue (Supplemental Figure S5C, http://links.lww.com/HC9/B984). The data indicate an accumulation of FAs in iCCA tissue due to increased synthesis.

### Fatty acid flux toward β-oxidation is impaired in iCCA tissue

**FIGURE 3:**
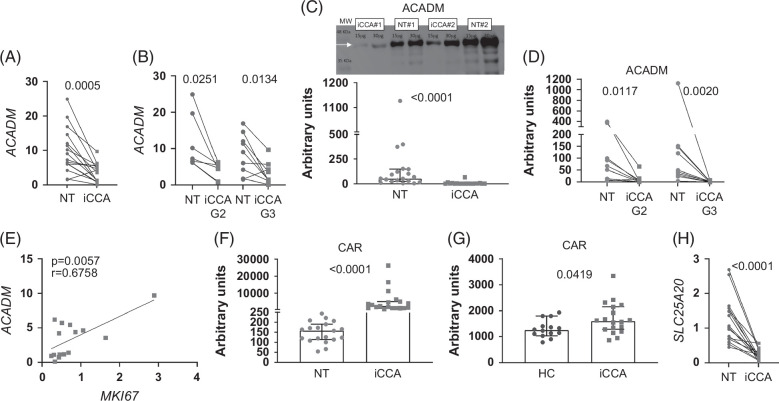
Fatty acid flux toward β oxidation is impaired in iCCA tissue. (A and B) Expression of the *ACADM* gene in NT, matched iCCA tissues (n=16), and in iCCA tissue stratified according to G stage (iCCA-G2=7; iCCA-G3=9). The paired *t* test was used to compare data. (C and D) ACADM protein level was measured in NT, matched iCCA tissues (n=19), and in iCCA tissue stratified according to G stage (iCCA-G2=9; iCCA-G3=10). Representative image showing a Western blot of ACADM (lane 1=standard ladders; lanes 2, 3, 6, and 7=iCCA tissues; lanes 4, 5, 8, and 9=NT tissues). Shown are medians and 95% CI. The Wilcoxon test was used to compare data. (E) The correlation between *ACADM* and *MKI67* expression was analyzed in 15 iCCA tissues. CAR content was quantified in NT, matched iCCA tissues (F; n=19), and in HC and iCCA-derived sera (G; HC=14; iCCA=18). Shown are medians and 95% CI. The Wilcoxon test was used to compare NT and matched iCCA tissue, whereas the Mann–Whitney *U* test was applied when comparing data from HC and iCCA-derived sera. (H) Expression of *SLC25A20* was measured in NT and matched iCCA tissues (n=17). The paired *t* test was used to compare data. Abbreviations: ACADM, acyl-CoA dehydrogenase medium chain; CAR, acylcarnitine; iCCA, intrahepatic CCA; MKI67, proliferation marker Ki-67; NT, non-tumoral.

FAs are an important fuel source for various tissues, being oxidized in mitochondria to provide energy. To investigate whether FAs accumulated in iCCA tissue are directed to β-oxidation, we analyzed the expression of *ACADM* (acyl-CoA dehydrogenase medium chain), which catalyzes the initial step of this pathway. *ACADM* transcription was lower in iCCA tissue than in NT tissue (Figure 3A) and, accordingly, the enzyme was about 25 times less expressed in iCCA compared with NT tissue (Figure 3C; Supplemental Figure S6, http://links.lww.com/HC9/B984). The same downregulation was observed in G2 and G3 tumors (Figures 3B, D). There was a positive correlation between the expression of *ACADM* and that of *MKI67* in iCCA (Figure 3E). Lipidomic analysis showed that in iCCA, there was a large accumulation of CAR, which participates in FA translocation to the mitochondria, indicating a profound impairment of FA flux toward β-oxidation (Figure 3F). This excess of CAR was mirrored in iCCA patients’ sera (Figure 3G). Of note, transcription of the solute carrier family 25 member 20 (*SLC25A20*), a mitochondrial-membrane–carrier protein involved in the transport of CAR into mitochondrial matrix for oxidation, was strongly impaired in iCCA tissue (Figure 3H). Our analyses compared iCCA tissue with adjacent NT tissue, mostly consisting of hepatocytes and not exclusively of normal bile ducts. We then compared gene expression of the most relevant genes related to lipid metabolism (*ACADM, ACACA*, and *FABP5*) in normal human cholangiocytes (NHC) with both NT and iCCA tissues. As shown in Supplemental Figure S7A, http://links.lww.com/HC9/B984, *ACADM* expression was downregulated in iCCA tissue compared with NT tissue but was upregulated when compared with NHC. *ACACA* expression was upregulated in iCCA compared with NT tissue but was unchanged when compared with NHC (Supplemental Figure S7B, http://links.lww.com/HC9/B984). The lipid transporter *FABP5* was higher in iCCA tissue than in NT tissue and NHC (Supplemental Figure 7C, http://links.lww.com/HC9/B984). In conclusion, comparison of iCCA with NHC indicates that lipid transport and oxidation are enhanced in cancer cells. The differential gene expression profile (*ACADAM*, *FABP5*, and *ACACA*) of iCCA compared with the hepatic microenvironment (NT) suggests that the liver plays a peculiar role in the metabolic reprogramming of iCCA.

**FIGURE 4:**
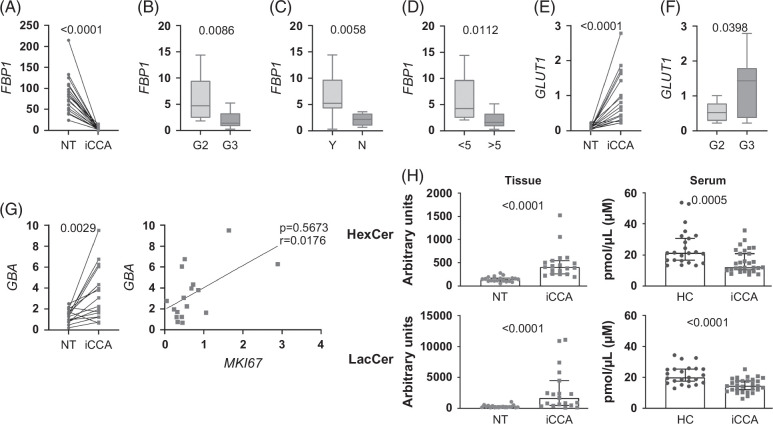
Glycolysis and pathways raising glucose content are enhanced in iCCA tissue. (A) Analysis of *FBP1* gene expression in NT, matched iCCA tissues (n=19) and in iCCA tissue stratified according to tumor G stage (B, iCCA-G2=8; iCCA-G3=11), to the presence of vascular invasion (C, Y=yes, n=7; N=no, n=10) and to tumor size in millimeters (D, iCCA<5, n=7; iCCA>5, n=10). Vascular invasion was histologically defined by tumor cells infiltrating vessel walls with associated thrombi, or intravascular cancer cells mixed with thrombi. Median, minimum, and maximum values are shown in the box and whiskers plots. The Wilcoxon test was used to compare NT and matched iCCA tissue, while the unpaired *t* test was used to compare other data. Expression of the *GLUT1* gene (E) was measured in NT and matched iCCA tissues (n=17) and in iCCA tissue stratified according to G stage (F, iCCA-G2=8, iCCA-G3=9). The Wilcoxon test was used to compare NT and matched iCCA tissue, while the unpaired *t* test was used to compare G2 and G3 tumors. Median, minimum, and maximum values are shown in the box and whiskers plots. (G, left panel) Expression of the *GBA* gene in NT and matched iCCA tissues (n=16). A paired *t* test was used. (G, right panel) The correlation between *ACACA* and *MKI67* expression was analyzed in 17 iCCA tumor tissues. (H) Analysis of the glycated Cers (HexCer and LacCer, upper and lower panels, respectively) in NT and matched iCCA tissues (n=19, left panel), and in HC-derived and iCCA-derived sera (iCCA=29, HC=23, right panel). Shown are medians and 95% CI. The Wilcoxon test was used to compare matched data, while the Mann–Whitney *U* test was used to compare unpaired data. Abbreviations: FBP1, fructose-1,6-bisphosphatase; GBA, glucocerebrosidase; HC, healthy control; HexCer, hexosylceramide; iCCA, intrahepatic cholangiocarcinoma; LacCer, lactosylceramides; MKI67, proliferation marker Ki-67; NT, non-tumoral.

We next explored the metabolic pathway of glycolysis, evaluating the transcription of fructose-1,6-bisphosphatase (*FBP1*), a rate-limiting enzyme that facilitates gluconeogenesis and inhibits glycolysis. *FBP1* was strongly repressed in iCCA, indicating activation of glycolysis (Figure 4A) mostly in G3 tumors, in those without vascular invasion and of larger size (Figures 4B–D). Glucose is made available by means of the GLUT1 transporter, whose transcription was enhanced in tumor specimens compared with NT tissue (Figure 4E) and in G3 tumors versus G2 tumors (Figure 4F). Glycated ceramides (hexosylceramide, HexCer, and lactosylceramides, LacCer) could represent an additional source of glucose by the action of the catabolic enzyme GBA (glucocerebrosidase). *GBA* transcripts were upregulated in iCCA (Figure 4G, left panel) and correlated with *MKI67* transcription (Figure 4G, right panel). Tumor content of HexCer and LacCer was increased compared with NT tissue (Figure 4I, left panels) while serum concentrations showed an opposite trend (Figure 4I, right panels). Our data indicate that β oxidation is poorly involved in energy fueling, while glycolysis and pathways to enhance glucose content are enhanced in iCCA.

### LDs are fewer in iCCA than in normal tissue and are detected mainly in M2 macrophages

**FIGURE 5:**
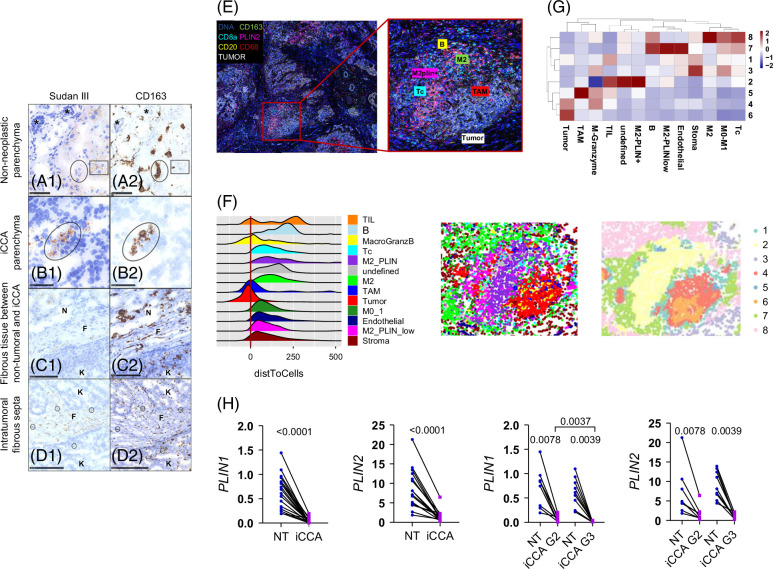
Lipid droplets, CD163+ M2 macrophage distribution, and perilipin expression in non-neoplastic parenchyma and in iCCA. (A1) In non-neoplastic parenchyma, small-sized and large-sized LDs (yellow-orange vacuoles) were detected in the cytoplasm of non-neoplastic cells, while bile ductular structures (asterisk) were negative. Hepatocytes also contained LDs (square). (A2) CD163 staining of A1 tissue identifies macrophages (circle) and hepatocytes (square) containing LDs. (B1) iCCA parenchyma showing a cluster of cells containing small-sized and medium-sized lipidic vacuoles. (B2) CD163 staining of B1 tissue highlights groups of LDs in the cytoplasm of CD163 immunopositive macrophages (circles). (C1) LDs distribution in fibrous bands (F) delimiting neoplastic growth between the liver parenchyma (N) and the tumor (K). The LDs are mainly detected adjacent to the tumor growing boundaries. (D1) Abundant, homogeneously distributed deposits of small-sized and medium-sized LDs mainly localized in the intratumoral fibrous bands (F) among intratumoral growing nodules (K). (C2 and D2) CD163 staining of C1 and D1 tissues. In C2, CD163+ cells containing LDs were found mainly next to iCCA cells. In D2, CD163+ immune cells containing LDs were identified in the fibrous part and sometimes intermingled with growing iCCA cells (circles). The images are the most representative of the 6 biopsies analyzed. (E) Spatial distribution of immune cell subsets within the iCCA tissue analyzed through Imaging Mass Cytometry. Marker expression profiles across all the acquired regions of interest (ROI) using MCD viewer visualization software are shown. A representative region of interest is shown at a higher magnification, together with its cell mask. (F) Shown is a distance plot of cell populations from the tumor patch. (G) Heatmap showing the 8 identified cell neighborhoods compositions and the relative cell type proportions *z*-score normalized per column (top). Cell masks colored by relative cell-neighborhood (bottom). (H) Expression of the perilipins *PLIN1* and *PLIN2* was measured in NT, matched iCCA tissues (n=17), and in iCCA tissue stratified according to G stage (iCCA-G2, n=8; iCCA-G3, n=9). The Wilcoxon test was used to compare matched data, while the Mann–Whitney *U* test was used to compare unpaired data. Abbreviations: iCCA, intrahepatic cholangiocarcinoma; NT, non-tumoral; PLIN, perilipins; TAM, tumor-associated macrophages; Tc, T cytotoxic.

LDs store excess intracellular FAs to avoid the generation of potentially toxic lipids.^23^ Through Sudan staining, small and large LDs were found to be homogeneously distributed in non-neoplastic parenchyma, and observed in the cytoplasm of both hepatocytes and Kupffer cells (CD163+ macrophages). They were absent in normal bile ducts (Figure 5A1, A2). In neoplastic tissue, there were small amounts of LDs and occasionally found in the cytoplasm of iCCA cells (Figure 5B1). Small amounts of LDs were also detected in the fibrous septa between non-neoplastic and neoplastic parenchyma, mainly adjacent to the tumor growing boundaries (Figure 5C1). Interestingly, LDs were more numerous and homogeneously distributed in the intratumoral fibrous septa (Figure 5D1). CD163 immunostaining identified small clusters of M2 macrophages containing LDs in the iCCA parenchyma (Figure 5B2). In the fibrous bands between non-neoplastic and neoplastic parenchyma, CD163+ LD-containing cells mainly accumulated close to iCCA cells (Figure 5C2), while they were more numerous and homogeneously distributed in the intratumoral fibrous septa (Figure 5D2). We investigated the distinct distribution patterns of immune cell (monocytes/macrophages and T/B cells) subsets within iCCA by Imaging Mass Cytometry (IMC). This technique enabled us to assess the spatial organization of immune cells within paraffin-embedded tissues by means of a deep multiparametric panel and to analyze the tumor microenvironment (TME) (Figure 5E). Our unsupervised spatial analysis unveiled a pronounced proximity of CD68+M0-1, CD163+perlipin2 (PLIN2)^low^ M2 macrophages and CD3+CD8+ T cytotoxic (Tc) cells to tumor regions (E-cadherin Pankeratin, ECAD+, PANK+). Instead, CD163+ M2 macrophages, CD163+PLIN2+ M2, and B cells (CD20+) were predominantly localized in the peritumoral zone (Figure 5F). We then characterized cellular “neighborhoods” (Figure 5G upper and lower panels), which refer to repetitive spatial arrangements of groups of cells within the TME, which may not be caught by single-cell interaction analysis. We identified 8 different neighborhoods. Tumor and stromal environments have been caught in single neighborhoods as expected. M2 PLIN2+ cells have shown interactions with tumor-infiltrating lymphocytes in Cluster Neighborhood (CN) 2. M2 PLIN2^low^ macrophages with B cells, endothelial cells, M0–M1 macrophages, and a small fraction of Tc were instead localized in CN7. Finally, CN8 highlighted an interaction between M0-1, M2 macrophages, Tc, and a small fraction of B cells. In keeping with the reduced amount of LDs in the iCCA parenchyma, transcriptional regulation of LD markers such as PLINs was impaired in iCCA samples, with *PLIN1* and *PLIN2* being significantly less expressed than in NT tissues (Figure 5H). Both G2 and G3 tumors downregulated *PLIN1* and *PLIN2* transcription and, of note, *PLIN1* transcription was further decreased in G3 tumors (Figure 5H). Our data indicated that tumor tissue displayed only few LDs, which were mainly localized in the fibrous septa and accumulated within M2 macrophages.

**FIGURE 6:**
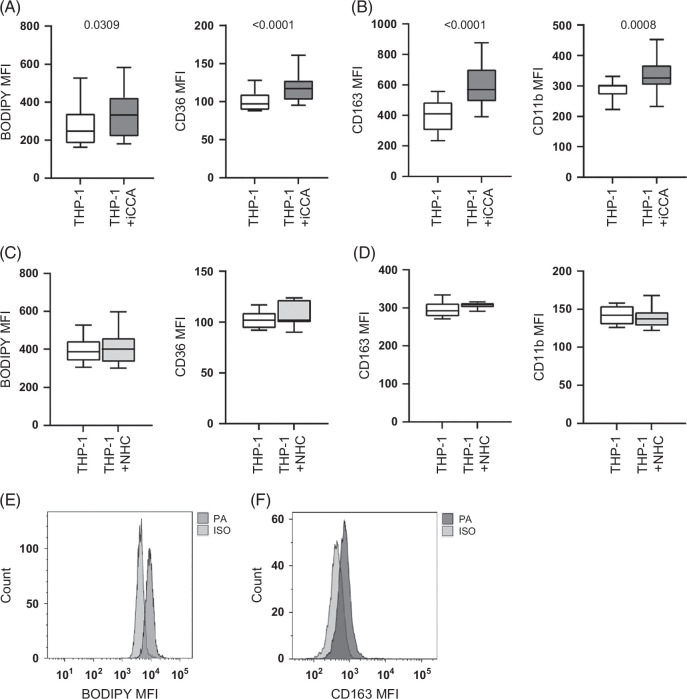
Increased lipid content and phenotype alterations in THP-1 monocytes co-cultured with iCCA cells. The lipid content was measured in THP-1 after 4 hours of co-culture with 7 primary iCCA cell cultures (A, left panel) or NHC cell cultures (C, left panel) as BODIPY 505/515 expression (MFI). The THP-1 phenotype was evaluated as MFI of CD36 (A and C, right panels), CD163 (B and D, left panels), and CD11b (B and D, right panels). Shown are medians and 95% CI. The Mann–Whitney *U* test was used to compare data. The BODIPY 505/515 (E) and CD163 (F) expression after treatment with 200 μM of PA or ISO is represented. Abbreviations: iCCA, intrahepatic cholangiocarcinoma; ISO, isopropanol; MFI, mean fluorescence intensity; NHC, normal human cholangiocyte; PA, palmitic acid.

Because of the peculiar localization of the LD-containing M2 macrophages next to iCCA cells, we tested the influence of tumor cells on TME lipid content by co-culturing primary iCCA cells with a monocyte cell line (THP-1). Interestingly, THP-1 cells showed an increased lipid and CD36 scavenger receptor expression (Figure 6A). Concomitantly, the expression of the M2 markers CD163 and CD11b was higher in THP-1 cells co-cultured with tumor cells (Figure 6B). In contrast, NHC had no effect on the lipid content of the monocyte cell line and on the expression of CD36 and M2 markers (Figures 6C, D). To assess whether cell–cell contact was required to drive these changes, we co-cultured THP-1 and primary iCCA cells in a Transwell system. No significant differences were observed besides a modest increase in lipid content and CD163 and CD11b expression on THP-1 cells (Supplemental Figure S8, http://links.lww.com/HC9/B984). Palmitic acid (PA) treatment raised the lipid content and CD163 expression (Figures 6E, F). These results suggest that, in the presence of tumor cells, monocytes modified their lipid content and phenotype, and that the same effect could be recapitulated in the presence of FA. In order to explore the association of lipid accumulation with M2 immunosuppression, we analyzed the influence of lipid-loaded THP-1 on T cell proliferation. Carboxyfluorescein diacetate succinimidyl ester (CFSE)-loaded peripheral blood mononuclear cells (PBMC) from healthy controls were stimulated with phytohemagglutinin-L (PHA) and co-cultured with THP-1 cells pretreated with exogenous FA. As shown in Supplemental Figure S9A, http://links.lww.com/HC9/B984, T cells co-cultured with lipid-loaded THP-1 exhibited slower proliferation compared with untreated THP-1 cells. Also, THP-1 co-cultured with primary iCCA cells determined a lower activation and proliferation of T cells in PBMC stimulated with anti-CD3/anti-CD28 (Supplemental Figure S9B, http://links.lww.com/HC9/B984), suggesting that lipids harness the phenotype of THP-1 cells toward immunosuppression.

### Fatty acid mobilization is directed to membrane remodeling and formation

**FIGURE 7:**
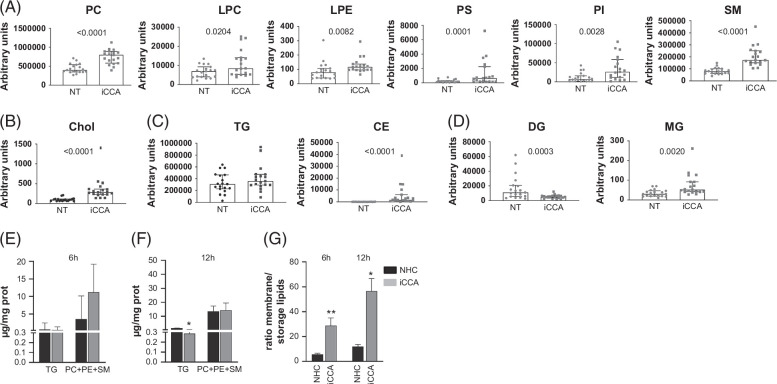
Quantitative metabolic flux assay indicates that fatty acid mobilization was directed to membrane remodeling and formation. Analysis of different membrane-forming lipid classes. PL species and SM (A) and Chol (B) in NT and matched iCCA tissues (n=19). (C, D) Analysis of the major neutral storage lipids in NT and matched iCCA tissues (n=19). Shown are medians and 95% CI. The Wilcoxon test was used to compare data. Analysis of storage (TG) and membrane-forming lipids PC, PE, and SM in NHC cell cultures and in primary iCCA cell cultures (n=3) at 6 (E) and 12 (F) hours. Panel (G) reports the membrane-to-storage lipids ratio. Shown are medians and 95% CI. The Mann–Whitney *U* test was used to compare data. Abbreviations: CE, cholesteryl esters; Chol, cholesterol; DG, diacylglycerols; iCCA, intrahepatic cholangiocarcinoma; LPC, lysophosphatidylcholine; LPE, lysophosphatidylethanolamine; MG, monoacylglycerol; NHC, normal human cholangiocyte; NT, non-tumoral; PC, phosphatidylcholine; PE, phosphatidylethanolamine; PI, phosphoinositide; PS, phosphatidylserine; SM, sphingomyelin; TG, triglyceride.

FAs provide essential lipids for membrane biogenesis and remodeling, thus supporting tumor proliferation. Indeed, PL and SPL are the main components of plasma membranes that directly derive from FAs. Several species belonging to these lipid classes were upregulated in iCCA compared with NT tissue, such as PC, lysophosphatidylcholine (LPC), lysophosphatidylethanolamine (LPE), phosphatidylserine (PS), phosphoinositides (PI), and SM (Figure 7A). Chol accumulation also supports iCCA membrane building (Figure 7B). Conversely, serum content showed an opposite trend (Supplemental Figure S10, http://links.lww.com/HC9/B984). Lipidomic analysis of the major storage lipids revealed that the TGs were equally distributed in iCCA and NT tissues, while CE was predominantly found in iCCA (Figure 7C). Diacylglycerols (DGs) were 3-fold less and monoacylglycerols (MGs) were 2-fold more abundant in iCCA (Figure 7D), suggesting high FA mobilization. Serum levels of TG and CE were unchanged (Supplemental Figure S11, http://links.lww.com/HC9/B984).

To further explore whether iCCA-derived FAs were mainly destined to membrane formation rather than to energy storage, we performed a quantitative metabolic flux investigation on NHC and primary iCCA cell cultures. Cells were incubated with palmitic acid d31 (PAD31) and its incorporation in complex lipids was inspected with high-resolution mass spectrometry after 6 or 12 hours. Among prevalent storage and membrane-forming lipids, TG, PC, phosphatidylethanolamine (PE), and SM were considered, respectively. In NHC and iCCA cell cultures, PAD31 was mainly incorporated into membrane lipids (Figures 7E, F). After 12 hours of incubation, the PAD31 incorporated in storage lipids (TG) was lower in tumor cells than in NH cultures (Figure 7F). Interestingly, at both times of incubation with PAD31 as FA precursor, the membrane-to-storage lipid ratio was significantly higher in iCCA than in NHC cultures (Figure 7G). Metabolic flux assay demonstrated that iCCA cells address FA to synthesize membrane-forming lipids.

### Fatty acids are precursors of bioactive lipids that regulate relevant cancer processes

**FIGURE 8:**
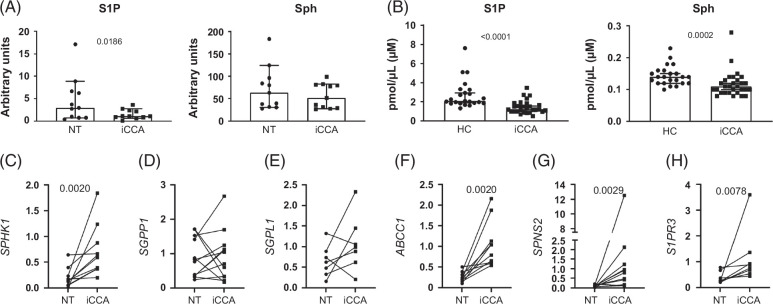
Increased synthesis and extrusion from the cells support the S1P autocrine role in iCCA tissues. Analysis of S1P and its precursor Sph in NT and matched iCCA tissues (A; n=11), and in HC and iCCA-derived sera (B; HC=23, iCCA=29). Shown are medians and 95% CI. The Wilcoxon test (A) or Mann–Whitney *U* test (B) was used to compare data. Gene expression analysis of the main S1P metabolizing enzymes (C, D, E), the main S1P transporters (F, G), and S1P receptor 3 (H) in NT and matched iCCA tissues (SPHK1, n=10; SGPP1, n=12; SGPL1, n=7; ABCC1, n=10; SPNS2, n=11; S1PR3, n=8). The Wilcoxon test (C–E, G, and H) or paired *t* test (F) was used to compare data. Abbreviations: ABCC1, ATP binding cassette subfamily C member 1; HC, healthy control; iCCA, intrahepatic cholangiocarcinoma; NT, non-tumoral; S1P, sphingosine-1-phosphate; S1PR3, sphingosine-1-phosphate receptor 3; SGPL1, sphingosine-1-phosphate lyase 1; SGPP1, sphingosine-1-phosphate phosphatase 1; Sph, sphingosine; SPHK1, sphingosine kinase 1; SPNS2, SPNS lysolipid transporter 2 sphingosine-1-phosphate.

In the SPL class, Cer and its downstream metabolite S1P are the main bioactive lipids, the latter acting as pro-survival and pro-proliferative mediators.^24^ S1P was decreased in iCCA tissue while its dephosphorylated precursor sphingosine (Sph) did not differ in iCCA compared with NT tissues (Figure 8A). In iCCA patients’ serum, they were about 50% and 20% less expressed, respectively (Figure 8B). Among several genes that regulate S1P content, mRNA expression of *SPHK1*, which plays a major role in phosphorylating Sph to produce S1P, was upregulated (Figure 8C). Conversely, sphingosine-1-phosphate phosphatase 1 (*SGPP1*), which transiently dephosphorylates S1P back to Sph, and the sphingosine-1-phosphate lyase 1 (*SGPL1*), which irreversibly cleaves S1P, were unchanged (Figures 8D, E). As for S1P extrusion from the cells, the transcript amount of the 2 transporters, the ATP binding cassette subfamily C member 1 (*ABCC1*) and the SPNS lysolipid transporter 2 sphingosine-1-phosphate (*SPNS2*), were both increased in iCCA tissue (Figures 8F, G). Once exported, S1P is capable of activating the sphingosine-1-phosphate receptors (S1PRs) expressed in the same cell in an autocrine manner.^24^,^25^ Interestingly, in iCCA tissue, *S1PR3* transcripts were higher than in NT tissue (Figure 8H).

## DISCUSSION

Cancer cells adapt their metabolism to sustain the intense rate of proliferation and the broad energy demand. Lipid dysmetabolism has been a matter of intense study over the last years due to the essential functions carried out by these bioactive mediators in tumor progression.^26^

In this study, we analyzed lipid metabolism rewiring in iCCA, a rare malignancy arising from the biliary tree, characterized by a globally increasing incidence and mortality rate. We observed that, predominantly unsaturated, FAs accumulated in iCCA compared with NT tissue, a finding mirrored by an increase in patients’ serum. Cancer cells convert toxic saturated into unsaturated FAs to reduce lipotoxicity that triggers apoptosis.^27^ Moreover, lipid biosynthesis and desaturation play a crucial role in HCC tumorigenesis and progression,^28^ whereas an increased monounsaturated/saturated FA ratio is associated with cancer invasiveness in various malignancies.^29^,^30^ We also observed an over 2-fold increase of FAHFA in iCCA biopsies. FAHFA is a novel family of branched FA esters of hydroxy fatty acids with anti-inflammatory properties, recently described by Yore et al.^31^ Interestingly, in human colon cancer cell lines as well as in tumor colon biopsies, FAHFA content is significantly elevated when compared with adjacent normal tissue, suggesting that FAHFA synthesis may occur as an escape mechanism of tumors from hydroxyacid-induced apoptosis.^32^ In addition, among 11 lipid species characterized by a conjunctive lipidomic approach, FAHFA were identified as potential biomarkers to distinguish patients with colorectal cancer from healthy subjects.^33^

An altered metabolome profile has been described in the sera of patients with iCCA.^6^ Of note, 9 specific metabolites among the mainly dysregulated lipid classes show higher diagnostic values than CA 19-9, which is the unspecific biomarker used in the diagnosis of CCA. We did not investigate the single lipid metabolite, but in agreement with this study, we found lower levels of SM and PC and elevated levels of BA classes in the serum from patients with iCCA.

In this study, we compared iCCA tissue with adjacent NT tissue, mostly composed of hepatocytes rather than normal bile ducts. The lipid metabolism of cancer cells could be influenced by surrounding tissues, though the ways in which cancer cells utilize extracellular lipids are often influenced by the peculiar characteristics of the tumors, such as activation of the PI3K/Akt axis and mTORC1 signaling.^34^,^35^ An in-depth analysis of such signaling mechanisms is beyond the scope of the present study. Instead, comparing tumor and liver gene expression could be greatly informative since the liver represents the physiological and notably permissive iCCA environment. While normal tissues often uptake lipids from circulating free FAs, many cancers reactivate de novo lipid synthesis by increasing the expression of several key enzymes.^26^,^36^ CCA seems to be independent of de novo FA synthesis by relying, instead, on extracellular FA availability though data on the role of de novo lipogenesis in CCA progression are controversial.^8^ However, we showed that in iCCA, the key enzyme in FA synthesis, *ACACA* was highly expressed, while the overall data on various FA transporters pointed to a weak involvement of the FA-regulated uptake pathway. Accordingly, we obtained conflicting data on macropinocytosis, a process involved in the nonselective uptake of extracellular molecules and nutrients that has been described in cancer cells for the assimilation of extracellular proteins and amino acids^22^,^37^ as well as for the active uptake of extracellular FAs.^21^ Cells dispose of the bulk of FAs to promote the β-oxidation process, to synthesize the core of LDs, to arrange membrane formation and remodeling, and to obtain bioactive mediators.^4^ In this study, we showed that iCCA seems poorly dependent on FA mitochondrial catabolism to meet energy demand. In fact, CAR accumulation together with a low expression of *ACADM* and *SLC25A20* suggests that β-oxidation could be impaired. Consistent with our findings, evidence gathered from humans and mice samples showed that a dysfunctional β-oxidation correlates with the accumulation of FAs and CAR and alters the patients’ lipid profile.^38^ In HCC, suppression of β-oxidation, as a result of either CPT2 or SLC25A20 downregulation, induced extensive storage of CAR that directly contributed to hepatocarcinogenesis.^39^,^40^ Of note, long-chain CARs were highly expressed in tumor tissue and related to the premature senescence of iNKT cells in HCC patients.^41^ In iCCA, we found a strict energy dependence on glucose, also evident in tumors lacking vascularization. The exacerbation of tumor hypoxia might favor anaerobic glycolysis as an energy supplier metabolism. Of note, we found an upregulation of *GBA* that makes glucose available to the cells by specifically removing it from glucosylceramide that accumulated in iCCA more than in NT tissue.

With respect to LDs, it is well established that they represent a reservoir of FAs as nontoxic neutral lipids (TG and CE) and that they regulate FA efflux to neighboring cells.^12^ We observed that LDs are poorly expressed in iCCA (both in tumor cells and TME), being more evident in the fibrotic perilesional areas, where cells of the immune system migrate, as previously described in the setting of melanoma.^42^ Indeed, LDs were abundant in M2 CD163+ cells accumulated next to iCCA cells and in the intratumoral fibrous septa. In the same regions, IMC analysis revealed that M2-PLIN^+^ macrophages interact with tumor-infiltrating lymphocytes, whereas M2-PLIN^low^ macrophages form neighborhoods with B cells. In the stromal compartment, the neighborhood analysis highlighted a neighborhood composed of M2, M1-0 macrophages, and Tc cells. A previous study provided evidence that, at the invading tumor edge of HCC tissues, B cells induce TAMs repolarization into M2 macrophages, creating favorable conditions for tumor growth.^43^ Of note, the lipid content in THP-1 cells expressing M2 markers and lipid transporters increased in the presence of iCCA cells or after stimulation with FA. We speculate that iCCA-derived LDs, together with B cells, might serve as a strategy for cancer immune escape. It has been reported that LD-dependent FA metabolism modulates the immune suppressive phenotype of TAM, promoting malignant progression.^16^ Differential abundance analysis between proteomes of liver-associated and HCC-associated macrophages showed that the LD-associated PLIN2 marker was strongly enriched in TAM.^44^ In iCCA, single-cell RNA-seq revealed increased frequencies of MARCO^high^ macrophages expressing PLIN2 and the FA-binding receptor FABP5 in tumoral compared with peritumoral tissues.^45^ However, the role of LDs in TAM and dendritic cells is still not fully established, although previous studies revealed that LD accumulation in dendritic cells resulted in impaired antigen cross-presentation in cancer.^18^

To maintain a high proliferative pace, iCCA mainly directs FAs to membrane formation and remodeling. In fact, we showed elevated PL, SM, and Chol content by mass spectrometry analysis and incorporation of FAs into PL instead of TG by metabolic flux analysis. In line with this, it has been shown that several cancers upregulate Chol and SPL biosynthesis in order to modulate membrane composition, strength, and dynamics.^46^ Among FA-derived sphingolipids, S1P plays a crucial role in cell proliferation and survival.^47^ Our data on SPHK1/S1P pathway activation are consistent with recently reviewed preclinical data.^9^ Notably, we found elevated tissue expression of *ABCC1* and *SPNS2* transporters that extrude S1P from the cells. In accordance, it has been reported that simultaneous high expression of *SPHK1* and *ABCC1* transporter mirrors S1P export associated with HCC progression.^48^ Interestingly, S1P acts on intracellular targets but it is also released in the extracellular compartment where it binds to G-protein coupled to S1P membrane receptors (S1PR1–5) thus activating autocrine and paracrine signaling.^49^ Indeed, we observed that *S1PR3* was highly expressed in iCCA compared with NT tissues, as described for lung and other cancers where activation of *S1PR3* mediates cell migration, invasion, and proliferation.^50^

In conclusion, this study provides evidence in support of FA accumulation being dependent upon impairment of β-oxidation. Furthermore, FAs promote iCCA aggressiveness by supporting membrane biogenesis and the generation of bioactive lipids that boost proliferation. Importantly, the predominance of unsaturated FAs protects cancer cells from saturated FA-derived lipotoxicity. In addition, FAs folded in CAR and LDs might be mobilized by iCCA in the TME as a strategy to support cancer immune escape. Overall, these data suggest that targeting FA metabolism could represent a viable innovative therapeutic intervention for iCCA.

## Supplementary Material
