# Supplementary material for: Cholangiocarcinoma cells direct fatty acids to support membrane synthesis and modulate macrophage phenotype
Source: Hepatol Commun. 2025 May 23;9(6):e0717. doi: 10.1097/HC9.0000000000000717 (PMC12106197; doi:10.1097/HC9.0000000000000717)
Supplement: Supplementary file 1 [file hc9-9-e0717-s001.docx]

**SUPPLEMENTARY INFORMATION**

**Cholangiocarcinoma cells direct fatty acids to support membrane synthesis and modulate macrophage phenotype.**

Supplementary Materials and Methods pages 2-5

Supplementary Tables pages 6-11

Supplementary Figures pages 12-23

**Supplementary Materials and Methods**

**Samples preparation for LC-MS and Western Blotting analysis.** iCCA and NT tissues (35-40 mg) were added by 100 μl of PBS with Protease Inhibitor (Complete™, Mini, EDTA-free Protease Inhibitor Cocktail, Roche) and thoroughly homogenized by a Tissue Lyser LT (Qiagen). Twenty microliters of each homogenate were destined to LC-MS analysis as detailed below. The remaining volume was centrifuged at 24000 x g for 30 minutes at 4° C and the supernatants collected for further BCA protein determination. 30 μg of protein’s extracts were separated by SDS-page electrophoresis and blotted onto a nitrocellulose membrane. Anti-ACADM (Abcam, ab92461, 1:10000 in TBS-T) antibody was used. GAPDH (Sigma, G9545, 1:5000 in TBS-T) expression was quantified for data normalization. The protein bands were detected by chemo-luminescent method by Alliance UVITEC (Cambridge, UK) which provided the band intensity quantification as well. Twenty-five microliters of serum added by 75 μl of PBS with Protease Inhibitor were processed for LC-MS analysis as detailed below.

**Lipid profile characterization by LC-MS/MS.** iCCA and NT tissue homogenate (20 μL, corresponding to less than 10 mg of tissue homogenate) or serum (25 μL) were diluted with PBS containing Protease Inhibitor to 100 μL, which were extracted with methanol/chloroform (850 μL, 2:1, v/v) with an oscillator thermo-mixer (30 min 5°C, 1000 RPM). After centrifugation (25 min at 20000 x g, 4°C) the organic phase was evaporated under a nitrogen stream. The residues were dissolved in 100 µl isopropanol/acetonitrile (2:1, v/v) + 0.5 mg BHT and withdrawn in a glass vial. Pure extracts (5 µL) were directly injected in LC-MS/MS. The LC-MS/MS consisted of a Shimadzu UPLC coupled with a Triple TOF 6600 Sciex (Concord, ON, CA) equipped with Turbo Spray IonDrive. All samples were analyzed in duplicate in both positive and negative mode with electrospray ionization. Spectra were contemporarily acquired by full-mass scan from 200-1500 m/z (100 ms accumulation time) and data-dependent acquisition from 50-1500 m/z (40 ms accumulation time, top-20 spectra per cycle 0.8 s). Declustering potential was fixed to 50 eV, and the collision energy was 35±15 eV. The chromatographic separation was reached on a reverse-phase Acquity CSH C18 column 1.7 μm, 2.1 × 100 mm (Waters, Franklin, MA, USA) equipped with a precolumn by a gradient between (A) water/acetonitrile (60:40) and (B) 2-propanol/acetonitrile (90:10), both containing 10-mM ammonium acetate and 0.1% of formic acid. The flow rate was 0.4 mL/min, and the column temperature was 45°C. The elution gradient (%B) was set as below: 0–2.0 min (40%), 2.0–2.5 min (40%–50%), 2.5–12.5 min (50%–55%), 12.5–13.0 min (55%–70%), 13.0–19.0 min (70%–99%), 19.0–24.0 min (99%), and 24.0–24.2 (99%–40%) and kept constant until 30 min [1].

**Cell cultures.** Normal human cholangiocyte (NHC) cells (kindly provided by professor JM Banales, University of the Basque Country (UPV/EHU), San Sebastian, Spain) were isolated from normal liver tissue specimens and maintained as previously described [2]. Primary tumor cell cultures were established from patients affected by iCCA as previously described [3]. Briefly, tumor samples were treated by enzymatic and mechanical dissociation with the human Tumor Dissociation Kit and gentleMACS Dissociator (Miltenyi Biotec), according to the manufacturer's instructions. The cell suspension was filtered and centrifuged to obtain a cell pellet that was plated in tissue culture flasks (Corning, NY, USA) with Dulbecco’s Modified Eagle Medium (Thermo Fisher Scientific) supplemented with 10% fetal bovine serum (FBS, HyClone, GE Healthcare, South Logan, Utah, USA), 1% antibiotic antimycotic solution (Merck) and 1% non-essential amino acids (Thermo Fisher Scientific). Cytokeratin19 (CK19) was evaluated by flow cytometry in a patient-derived primary iCCA cell culture used in co-cultured experiments and for the metabolic flux analysis (Supplementary figure 12). Briefly, iCCA cells were fixed with BD Cytofix/Cytoperm (BD Biosciences, San Diego, CA, USA) and permeabilized with the BD Perm/Wash buffer (BD Biosciences) in the presence of the FITC anti-Cytokeratin 19 mouse monoclonal antibody (clone SB39g, Abcam, Cambridge, UK) for 30 min at 4°C, according to the manufacturer’s instructions. The human monocyte cell line THP-1 was maintained in RPMI-1640 supplemented with 10% FBS, 1% antibiotic antimycotic solution and 1% Glutamine (Merck).

**Co-cultures and palmitic acid treatment.** Cells derived from 7 primary tumor cell cultures were plated at 5x10^4^ cells/well in a 24 wells plate (Corning Costar, Glendale, USA). After their adhesion, the medium was removed, cells accurately washed with PBS and THP-1 cells added (5x10^5^ cells/well) in RPMI w/o serum. After 4h THP-1 cells were harvested, washed with PBS, labeled with BODIPY™505/515 (4,4-Difluoro-1,3,5,7-Tetramethyl-4-Bora-3a,4a-Diaza-s-Indacene, Thermo Fisher) at 2μM for 5min at 37°C and washed with PBS 2% FBS. To evaluate the THP-1 phenotype, cells were also labeled with anti-CD45 BV421, anti-CD36 PE, anti-CD11b BB515 and anti-CD163 Alexa Fluor 647 (all by BD Biosciences). The fluorescence of BODIPY™ 505/515 and the marker expression were analyzed by using a 12-color FACSCelesta (BD Biosciences) flow cytometer. Kaluza™ software (Beckman Coulter, Brea, CA, USA) was used for data analysis. In the Transwell® experiment, NHC or primary iCCA cell cultures (5x10^4^) were plated in a six well Transwell® plate (Corning) and THP-1 cells were added in the inserts, all in RPMI w/o serum. After 4h, THP-1 were harvested and labeled with BODIPY™505/515, CD36, CD11b and CD163. For the treatment with FA, 200 μM PA (Merck) was used. It was diluted in PBS with 14% of BSA, warmed at 55°C for 10 min and added to RPMI w/o serum. THP-1 cells (5x10^5^/well) were treated for 4 h at 37°C with PA or with isopropanol (prepared as PA) as negative control. After incubation, THP-1 cells were harvested and labeled with BODIPY™505/515, anti-CD163 and anti-CD36. The same conditions of THP-1 treatment were used in proliferation assays. PBMC from healthy controls were labelled with Carboxyfluorescein Diacetate Succinimidyl Ester (CFSE) according to the manufacturer's instructions and seeded at 2,5x10^5^ cells/well in the presence of PA-treated or untreated THP-1 cells (1x10^5^ cells/well) in complete medium containing 20ng/ml of IL-2 (Peprotech) and 10 ug/ml Phytohemagglutinin-L (PHA-L, Roche). Unstimulated and PHA-stimulated PBMC without THP-1 were used as negative and positive controls. After 3 days of co-culture, cells were harvested, labelled with anti-CD3 APC-H7 and their proliferation analysed by flow cytometry. T cell proliferation was also measured in CFSE-labeled PBMC stimulated for 3 days with anti-CD3/anti-CD28 (ImmunoCult™ Human CD3/CD28 T Cell Activator, StemCell Technologies) and IL-2 stimuli in the presence of THP-1 or THP-1 co-cultured with iCCA primary cells. CD69 PE (BD Bioscience) was used to identify the activated T cells.

**Metabolic flux analysis.** 1×10^5^ cells derived from low-passage primary tumor cell culture and 5×10^4^ NHC and were plated in 12 wells plates in DMEM/10%FBS/1% essential amino acids and RPMI/10% FBS, respectively. After 72h, cells were conditioned with serum-deprived medium for two hours in order to remove exogenous lipids [4]. The medium was then replaced with a serum-free medium containing 100 µM PAD31 (deuterated d31 palmitic acid) in FA-free BSA for a further 6 or 12 hours. The cells were then collected, washed twice in PBS and finally pelleted at 500 x g, 4°C for 5 minutes. Then***,*** cell lipids were extracted with methanol/chloroform and the extracts analyzed by high-resolution mass spectrometry using an untargeted data-dependent approach (see paragraph Lipid profile characterization by LC-MS/MS). This way we monitored the incorporation of PAD31 in the structure of different lipids. The mass shift of the mass precursor depends on the number of PAD31 in each molecule which were, respectively, +31.19, +62.38, +93.57 Da. To create a library we firstly annotated natural lipids which incorporate at least 1 PA and their MS/MS spectra clearly confirmed its presence (Supplementary Table 3). Then we calculated mass shift for PAD31-labeled lipids and checked their presence in the spectrum of different cell extracts (either primary tumor cell cultures or NHC, Supplementary Table 4). This operation allowed us to determine the predominant lipid species for each subclass in order to create a final library containing only PAD31-labeled lipids.

**Quantitative analysis of fatty acid-labeled lipids*.*** The dry pellets were then splitted in two aliquots for two different lipid analyses: (1) glycerolipids, sterol and FAs and (2) SPLs. Quantification of lipid species was performed after semi-automatic peak integration (Sciex OS ver 3.0, Sciex) and area interpolation using spiked-in deuterated standards at a pre-established concentration (150 ng, EquiSPLASH labeled standard, Avanti Polar Lipids, 330731, Alabaster, AL, USA, Supplementary Table 5) [5]. Lipid concentration (ng) then were normalized for each sample to their amount of protein content. Concentrations were expressed as ng/mg prot. Data were filtered excluding species which can also be found in non-labelled samples or in blank.

**Total lipid analysis.** Cells (50-100μg proteins) were extracted with methanol/chloroform (850 μL, 2:1, v/v) with an oscillator thermo-mixer (30 min 5°C, 1000 RPM). After centrifugation (25 min at 20000xg, 4°C) the organic phase was evaporated under a nitrogen stream. The residues were dissolved in 100 µl of isopropanol/acetonitrile (2:1, v/v) + 0.5 mg BHT and withdrawn in a glass vial. Pure extracts (5 µL) were directly injected in LC-MS/MS (see paragraph Lipid profile characterization by LC-MS/MS)

**SPL analysis*.***  Cells (50-100 μg proteins) were extracted in the same way as already reported with the addition of the alkaline methanolysis step (75 µL alcoholic KOH 1M, 2h at 38°C). After that, samples were neutralized by acetic acid (4 µL), centrifuged (25 min at 20000 xg, 4°C), and the organic phase was vacuum-evaporated. The residues were dissolved in 100 µL of methanol + 0.5 mg/mL BHT and withdrawn in a glass vial after an additional centrifugation. Pure extracts (5 µL) were directly injected in LC-MS/MS (see paragraph Lipid profile characterization by LC-MS/MS).

**Histochemistry and immunohistochemistry.** Serial 3 μm tissue sections from cryostatic iCCA tissues were stained with Sudan III to highlight LDs and then digitalized with Nanozoomer Hamamatsu and analyzed with the NDP.view software. Immunohistochemistry for CD163 (Roche Diagnostic) was performed on the same sections to visualize the M2 polarized macrophages. Immunohistochemistry was performed with the automatic immunostainer DAKO OMNIS (DAKO). Analyzes have been carried out on six patients. Slides were digitalized again and then evaluated by two pathologists.

**Imaging Mass Cytometry staining and acquisition.** Tissue slides were incubated with preheated 10% antigen retrieval solution (Dako) at 96°C for 30 minutes. After incubation, slides were cooled down at 70°C for 20 minutes and subsequently washed in Maxpar Water and Maxpar PBS (StandardBioTools, USA). The sections have been encircled with an A-PAP pen (BioOptica, Italy), and blocked with 3% BSA-Maxpar PBS solution for 45 minutes at room temperature to prevent nonspecific binding. Following blocking, the tissues were left in incubation overnight at 4°C with an antibody cocktail (antibodies to: ECAD, CD45, CD68, CD163, CD3, CD4, CD8 and CD20) prepared with 0.5% BSA-PBS solution. The slide was stained with a 1:400 dilution of Intercalator-Ir (StandardBioTools) in Maxpar PBS for 30 minutes at room temperature to label the nuclei after washes in 0.2% Triton X-100 in Maxpar PBS for 8 minutes, followed by 2 washes in Maxpar PBS for 8 minutes each followed by an air drying period. Sections were acquired using imaging IMC (Hyperion, StandardBioTools, USA). The regions of interest were designed following morphological evaluation on hematoxylin eosin staining.

**Imaging Mass Cytometry data analysis.** Previous to the analysis a quality check of the staining has been conducted through MCD Viewer software. After the quality check, through the Steinbock Toolkit [6] we generated the probability map via Ilastik v1.4.0 and a segmentation mask, based on the previous probability map, through CellProfiler v4.1.3. Cells were then phenotyped through an unsupervised approach by PhenoGraph clustering, of the Rphenograph package, with a nearest neighbour k value of 30. To evaluate cell distances, we used the minDistToCells function of the R package imcRtools (v1.8.0), assigning to each cell a distance value from tumor cells. Cell neighborhood clusters were constructed following the method proposed by Schürch et al. [7] with a k = 20 for the buildSpatialGraph function and a k = 8 clusters for the evaluation of the neighborhood cell type composition.

**References.**

1. Morano C, Roda G, Paroni R, Dei Cas M. Tip-tip filtration ameliorates single-phase extraction methods for plasma large-scale lipidomics analysis. *J Chromatogr B Analyt Technol Biomed Life Sci*. 2022;1189:123099.
2. **Ruiz de Gauna M, Biancaniello F**, González-Romero F, et al. Cholangiocarcinoma progression depends on the uptake and metabolization of extracellular lipids. *Hepatology*. 2022;76(6):1617-1633.
3. Oliviero B, Varchetta S, Mele D, et al. MICA/B-targeted antibody promotes NK cell-driven tumor immunity in patients with intrahepatic cholangiocarcinoma. *Oncoimmunology*. 2022;11(1):2035919. Published 2022 Feb 21.
4. Planas-Serra L, Launay N, Goicoechea L, et al. Sphingolipid desaturase DEGS1 is essential for mitochondria-associated membrane integrity. *J Clin Invest*. 2023;133(10):e162957. Published 2023 May 15.
5. Pagura L, Dumoulin PC, Ellis CC, et al. Fatty acid elongases 1-3 have distinct roles in mitochondrial function, growth, and lipid homeostasis in Trypanosoma cruzi. *J Biol Chem*. 2023;299(6):104715.
6. Windhager J, Zanotelli VRT, Schulz D, et al. An end-to-end workflow for multiplexed image processing and analysis. Nat Protoc. 2023;18(11):3565-3613.
7. Schürch CM, Bhate SS, Barlow GL, et al. Coordinated Cellular Neighborhoods Orchestrate Antitumoral Immunity at the Colorectal Cancer Invasive Front [published correction appears in Cell. 2020 Oct 29;183(3):838. doi: 10.1016/j.cell.2020.10.021]. Cell. 2020;182(5):1341-1359.e19.

| \| **Supplementary Table 1.**  **Characteristics of patients.** \| **iCCA** \| **%** \| \| --- \| --- \| --- \| \| Number of patients \| 27 \| - \| \| Female/Male \| 11/16 \| 40.7/59.3 \| \| Median age (years) - range \| 71.0 (57-82) \| - \| \| Liver cirrhosis: yes/not; na \| 2/23; 2 \| 7.4/85.2; 7.4 \| \| Vascular invasion: yes/not; na \| 14/11; 2 \| 51.9/40.7; 7.4 \| \| Perineural invasion: yes/not; na \| 2/23; 2 \| 7.4/85.2; 7.4 \| \| Lymph node status: N0  N1  Nx  na \| 18  4  4  1 \| 66.7  14.8  14.8  3.7 \| \| Number of tumors: single/multiple; na \| 7/18; 2 \| 25.9/66.7; 7.4 \| \| Tumor size: <5cm \| 10 \| 37 \| \| >5cm \| 14 \| 51.9 \| \| na \| 3 \| 11.1 \| \| Tumor stage: T1 \| 9 \| 33.3 \| \| T2 \| 17 \| 63 \| \| T3 \| 1 \| 3.7 \| \| T4 \| 0 \| 0 \| \| Tumor grade: G1  G2  G3  na \| 0  11  16  0 \| 0  40.7  59.3  0 \| \| **Risk factors** \|  \|  \| \| HBV \| 2 \| 7.4 \| \| HCV \| 1 \| 3.7 \| \| MASLD \| 7 \| 25.9 \| \| PSC \| 0 \| 0 \| \| Alcohol abuse \| 0 \| 0 \| \| Unknown \| 17 \| 63.0 \| \| DM: yes/no; na \| 8/14; 5 \| 29.6/51.8; 18.6 \| \| Dyslipidemia, Y/N, na  BMI Kg/m^2^, median, range  BMI ≤ 24.9/ >24.9; na \| 16/9; 2  25.3 (18.40-33.10)  10/12; 5 \| 59.2/33.3; 7.5  -  37/44.4; 18.6 \| \| TG, (mg/dL), median, range  TC, (mg/dL), median, range  LDL-C, (mg/dL), median, range  HDL-C, (mg/dL), median, range \| 112.5 (70-024)  194.0 (129-237)  117.0 (59-157)  53.5 (22-76) \| -  -  -  - \|   na, not available; G1, well differentiated; G2, moderately differentiated; G3, poorly differentiated; HBV, hepatitis B virus; HCV, hepatitis C virus; MASLD, metabolic dysfunction–associated steatotic liver disease; PSC, Primary sclerosing cholangitis; DM, diabetes mellitus type2; TG, triglycerides; TC, total cholesterol; LDL-C, low-density lipoproteins cholesterol; HDL-C, high-density lipoprotein cholesterol. |
| --- | --- | --- | --- | --- | --- | --- | --- | --- | --- | --- | --- | --- | --- | --- | --- | --- | --- | --- | --- | --- | --- | --- | --- | --- | --- | --- | --- | --- | --- | --- | --- | --- | --- | --- | --- | --- | --- | --- | --- | --- | --- | --- | --- | --- | --- | --- | --- | --- | --- | --- | --- | --- | --- | --- | --- | --- | --- | --- | --- | --- | --- | --- | --- | --- | --- | --- | --- | --- | --- | --- | --- | --- | --- | --- | --- | --- | --- | --- | --- | --- | --- |
|  |

| **Supplementary Table 2.**  **List of the primers used.** |
| --- |
| \| **Name** \| **Sequence 5’-3’** \| \| --- \| --- \| \| HPRT1 forward \| TGACACTGGCAAAACAATGCA \| \| HPRT1 reverse \| GGTCCTTTTCACCAGCAAGCT \| \| ACACA forward \| GCTGCTCGGATCACTAGTGAA \| \| ACACA reverse \| TTCTGCTATCAGTCTGTCCAG \| \| FABP5 forward \| ATACATGAAGGAGCTAGGAGTGG \| \| FABP5 reverse \| CTGAACCAATGCACCATCTGT \| \| FABP4 forward \| AAACTGGTGGTGGAATGCGT \| |
| \| SLC25A20 forward \| GAGCTGATCCGGGATGAAGG \| \| --- \| --- \| \| SLC25A20 reverse \| AAGGAAACAGGCCGCATTGG \| \| GLUT1 forward \| TTCACTGTCGTGTCGCTGTTT \| \| GLUT1 reverse \| ACGATGAACCATGGGATGGG \| \| PLIN1 forward \| AGGGAAGAAGTTGAAGCTTGAGG \| \| PLIN1 reverse \| TTCTGGAAGCATTCGCAGGT \| \| PLIN2 forward \| GCTGCAGTCCGTCGATTTCT \| \| PLIN2 reverse \| CCACACTCGGTTGTGGATCA \| \| SPHK1 forward \| AGGCTGAAATCTCCTTC \| \| SPHK1 reverse \| GTCTCCAGACATGACCACCAG \| \| SGPP1 forward \| TGGTCAAGTTGGAGGTCTTCT \| \| SGPP1 reverse \| CATATATAAGAGGGTACTGCCAGC \| \| ABCC1 forward \| TGCATCGTTCTGTTTGCTGC \| \| ABCC1 reverse \| TCAAGTACGTGGTGACCTGC \| |
| \| ZIP4 forward \| GACTGAGCCCAGAGTTGAGG \| \| --- \| --- \| \| ZIP4 reverse \| CAACTCGTGGCAGAACACG \| |
| \| DNM2 forward \| CCGCTGGTCAACAAACTGC \| \| --- \| --- \| \| DNM2 reverse \| CGGGTGACGATTCCTGAACC \| |
| \| SDC1 forward \| CTGCCGCAAATTGTGGCTAC \| \| --- \| --- \| \| SDC1 reverse \| TGAGCCGGAGAAGTTGTCAGA \| |
|  |

|  |
| --- |
|  |
|  |
|  |
|  |
|  |

**Supplementary Table 3.** List of natural lipids containing at least 1 palmitic acid analyzed by high resolution mass spectrometry.

| **Lipid name** | **Molecular mass** (m/z) | **Rt** (min) |
| --- | --- | --- |
| CAR 16:0 | 400.34003 | 2.61 |
| Cer 34:0;2O\|Cer 18:0;2O/16:0 | 540.53851 | 15.317 |
| Cer 34:1;2O\|Cer 18:1;2O/16:0 | 538.51416 | 15.08 |
| DG 30:0\|DG 14:0_16:0 | 558.51031 | 15.57 |
| DG 32:0\|DG 16:0_16:0 | 586.53595 | 16.134 |
| DG 32:1\|DG 16:0_16:1 | 584.52356 | 15.686 |
| DG 34:0\|DG 16:0_18:0 | 614.56775 | 16.624 |
| DG 34:1\|DG 16:0_18:1 | 612.55048 | 16.186 |
| DG 34:2\|DG 16:0_18:2 | 610.539 | 15.779 |
| DG 38:6\|DG 16:0_22:6 | 658.54095 | 15.54 |
| HexCer 34:1;2O\|HexCer 18:1;2O/16:0 | 700.57159 | 14.458 |
| LPC 16:0 | 496.34012 | 2.525 |
| LPC O-16:0 | 482.3605 | 3.387 |
| LPE 16:0 | 454.29156 | 3.018 |
| PC 30:0\|PC 14:0_16:0 | 706.53448 | 14.164 |
| PC 32:0\|PC 16:0_16:0 | 734.56427 | 14.915 |
| PC 32:1\|PC 16:0_16:1 | 732.55066 | 14.32 |
| PC 34:0\|PC 16:0_18:0 | 762.59637 | 15.539 |
| PC 34:1\|PC 16:0_18:1 | 760.58234 | 15.01 |
| PC 36:3\|PC 16:0_20:3 | 784.58173 | 14.619 |
| PC 36:4\|PC 16:0_20:4 | 782.56555 | 14.45 |
| PC 38:5\|PC 16:0_22:5 | 808.58405 | 14.528 |
| PC 38:6\|PC 16:0_22:6 | 806.56586 | 14.281 |
| PE 32:0\|PE 16:0_16:0 | 692.52106 | 15.122 |
| PE 34:1\|PE 16:0_18:1 | 718.53601 | 15.179 |
| PE P-32:0\|PE P-16:0_16:0 | 676.52673 | 15.442 |
| PE P-34:0\|PE P-18:0_16:0 | 704.55981 | 15.991 |
| PE P-34:1\|PE P-16:0_18:1 | 702.53851 | 15.495 |
| PE P-36:4\|PE P-16:0_20:4 | 724.52625 | 14.953 |
| SM 34:1;2O\|SM 18:1;2O/16:0 | 703.57178 | 14.088 |
| TG 44:0\|TG 14:0_14:0_16:0 | 768.7049 | 18.117 |
| TG 46:1\|TG 14:0_16:0_16:1 | 794.71924 | 18.156 |
| TG 48:0\|TG 16:0_16:0_16:0 | 824.76379 | 18.636 |
| TG 48:1\|TG 16:0_16:0_16:1 | 822.75073 | 18.416 |
| TG 48:2\|TG 16:0_16:1_16:1 | 820.73438 | 18.2 |
| TG 50:0\|TG 16:0_16:0_18:0 | 852.7973 | 18.865 |
| TG 50:1\|TG 16:0_16:0_18:1 | 850.78229 | 18.649 |
| TG 50:2\|TG 16:0_16:1_18:1 | 848.76624 | 18.437 |
| TG 52:0\|TG 16:0_18:0_18:0 | 880.82886 | 19.077 |
| TG 52:1\|TG 16:0_18:0_18:1 | 878.81232 | 18.87 |
| TG 52:2\|TG 16:0_18:1_18:1 | 876.79584 | 18.653 |
| TG 54:5\|TG 16:0_18:1_20:4 | 898.77893 | 18.34 |
| TG 56:6\|TG 16:0_18:1_22:5 | 924.79449 | 18.372 |
| TG 56:7\|TG 16:0_18:1_22:6 | 922.77948 | 18.269 |
| TG 58:0\|TG 16:0_18:0_24:0 | 964.92096 | 19.585 |
| TG 58:1\|TG 16:0_24:0_18:1 | 962.90436 | 19.434 |
| TG 58:2\|TG 16:0_18:1_24:1 | 960.88824 | 19.266 |
| TG 60:10\|TG 16:0_22:5_22:5 | 972.79468 | 18.103 |
| TG 60:11\|TG 16:0_22:5_22:6 | 970.78156 | 17.981 |

**Supplementary Table 4.** List of lipids containing in their structure at least 1 labelled palmitic acid (PAD31, indicated in the lipid abbreviation as 16:0d) analyzed by high resolution mass spectrometry under ESI positive conditions.

| **Labelled lipid** | **Molecular mass** (m/z) | **Rt** | **MS/MS** | **MS/MS** | **MS/MS** |
| --- | --- | --- | --- | --- | --- |
| CAR 16:0d | 431.5322 | 2.52 | 85.02 | 372.46 |  |
| DHCer 16:0d | 571.7307 | 15.22 | 266.26 | 553.73 |  |
| Cer 16:0d | 569.7064 | 14.97 | 264.27 | 551.703 |  |
| DG 14:0_16:0d | 589.7025 | 15.5 | 344.47 | 554.66 |  |
| DG 16:0d_16:0 | 617.7282 | 16.12 | 344.46 | 582.69 |  |
| DG 16:0d_16:1 | 615.7158 | 15.63 | 344.42 | 580.68 |  |
| DG 16:0d_18:0 | 645.76 | 16.59 | 344.46 | 610.72 |  |
| DG 16:0d_18:1 | 643.7427 | 16.12 | 344.46 | 608.7 |  |
| DG 16:0d_18:2 | 641.7312 | 15.72 | 344.46 | 606.69 |  |
| DG 16:0d_22:6 | 689.7332 | 15.52 | 344.47 | 432.09 | 672.7 |
| HexCer 16:0d | 731.7638 | 14.32 | 264.27 | 551.69 |  |
| LPC 16:0d | 527.5323 | 2.67 | 184.07 | 104.1 | 509.52 |
| LPC O-16:0d | 513.5527 | 3.18 | 184.07 | 104.1 |  |
| LPE 16:0d | 485.4838 | 2.87 | 344.46 |  |  |
| PC 14:0_16:0d | 737.7267 | 14.06 | 184.07 |  |  |
| PC 16:0d_16:0 | 765.7565 | 14.8 | 184.07 |  |  |
| PC 16:0d_16:1 | 763.7429 | 14.22 | 184.07 |  |  |
| PC 16:0d_18:0 | 793.7886 | 15.46 | 184.07 |  |  |
| PC 16:0d_18:1 | 791.7745 | 14.87 | 184.07 |  |  |
| PC 16:0d_20:3 | 815.7739 | 14.59 | 184.07 |  |  |
| PC 16:0d_20:4 | 813.7578 | 14.35 | 184.07 |  |  |
| PC 16:0d_22:5 | 839.7763 | 14.37 | 184.07 |  |  |
| PC 16:0d_22:6 | 837.7581 | 14.19 | 184.07 |  |  |
| PE 16:0d_16:0 | 723.7133 | 14.47 | 361.25 | 582.49 |  |
| PE 16:0d_18:1 | 749.7282 | 15.02 | 361.27 | 608.52 |  |
| PE P-16:0_16:0d | 707.7189 | 15.39 | 344.46 | 566.68 | 663.45 |
| PE P-18:0_16:0d | 735.752 | 15.92 | 344.46 | 594.73 |  |
| PE P-16:0d_18:1 | 733.7307 | 15.41 | 344.46 | 592.53 |  |
| PE P-16:0d_20:4 | 755.7185 | 14.52 | 368.36 | 614.7 | 570.54 |
| SM 16:0d | 734.764 | 13.99 | 184.06 |  |  |
| TG 14:0_14:0_16:0d | 799.8971 | 18.1 | 554.66 | 270.42 |  |
| TG 14:0_16:0d_16:1 | 825.9114 | 18.04 | 521.45 |  |  |
| TG 16:0_16:0_16:0d | 855.956 | 18.60 | 582.69 | 551.5 |  |
| TG 16:0_16:0d_16:1 | 853.9429 | 18.31 | 549.48 | 580.66 |  |
| TG 16:0d_16:1_16:1 | 851.9266 | 18.11 | 547.47 | 580.66 |  |
| TG 16:0d_16:0_18:0 | 883.9895 | 18.80 | 610.72 | 582.69 | 579.54 |
| TG 16:0d_16:0_18:1 | 881.9745 | 18.54 | 608.7 | 577.51 | 582.69 |
| TG 16:0d_16:1_18:1 | 879.9584 | 18.30 | 608.7 | 575.5 | 580.67 |
| TG 16:0d_18:0_18:0 | 912.0211 | 18.49 | 608.71 | 612.88 |  |
| TG 16:0d_18:0_18:1 | 910.0045 | 18.81 | 605.54 | 636.73 |  |
| TG 16:0d_18:1_18:1 | 907.988 | 18.63 | 608.7 | 603.53 |  |
| TG 16:0d_18:1_20:4 | 929.9711 | 18.32 | 625.51 | 608.71 |  |
| TG 16:0d_18:1_22:5 | 955.9867 | 18.36 | 651.58 | 656.7 |  |
| TG 16:0d_18:1_22:6 | 953.9717 | 18.25 | 608.71 | 649.44 |  |
| TG 16:0d_18:0_24:0 | 996.1132 | 19.50 | 691.79 | 695.62 |  |
| TG 16:0d_24:0_18:1 | 994.0966 | 19.40 | 689.63 | 608.7 |  |
| TG 16:0d_18:1_24:1 | 992.0804 | 19.20 | 687.62 | 692.79 | 608.71 |
| TG 16:0d_22:5_22:5 | 1003.987 | 18.03 | 699.53 | 656.7 |  |
| TG 16:0d_22:5_22:6 | 1001.974 | 17.92 | 697.52 | 656.7 |  |

**Supplementary Table 5.** Mass spectrometry conditions for Avanti Equisplash® Mix (#330731, Avanti Polar Lipids) used as quantitative reference for fluxomics.

| **Nome** | **ESI** | **Q1 (m/z)** | **Q3 (m/z)** | | | |
| --- | --- | --- | --- | --- | --- | --- |
| PC d7 | + | 753.6142 | 184.0738 | 482.3239 | 124.9996 |  |
| LPC d7 | + | 529.3998 | 184.0735 | 104.1071 | 346.3310 |  |
| PE d7 | + | 711.5669 | 570.5483 | 315.3382 | 272.2964 |  |
| LPE d7 | + | 487.3529 | 346.3337 | 315.3386 | 272.2967 |  |
| PG d7 | + | 759.5877 | 570.5484 | 346.3300 | 272.2951 |  |
| PI d7 | + | 847.6036 | 570.5472 | 346.3316 | 225.2194 |  |
| PS d7 | + | 755.5563 | 570.5479 | 359.3310 | 299.2593 |  |
| TAG d7 | + | 829.7977 | 570.5476 | 523.4723 | 225.2212 | 272.2964 |
| DAG d7 | + | 605.5842 | 299.2588 | 346.3342 | 570.5482 |  |
| MAG d7 | + | 381.3702 | 272.2975 | 346.3355 | 135.1170 |  |
| CE d7 | + | 675.6773 | 369.3519 | 161.1320 | 147.1165 |  |
| SM d7 | + | 738.6477 | 184.0738 | 720.6351 | 124.9995 |  |
| Cer d7 | + | 531.5477 | 271.3131 | 513.5385 | 289.3235 | 259.3126 |
| PE d7 | - | 709.5524 | 288.2938 | 241.2162 | 438.2582 |  |
| LPE d7 | - | 485.3378 | 288.2932 | 196.0383 |  |  |
| PG d7 | - | 740.5465 | 288.2934 | 241.2179 |  |  |
| PI d7 | - | 829.6132 | 241.0123 |  |  |  |
| PS d7 | - | 753.5415 | 667.5154 | 241.2181 | 377.2107 |  |
| Cer d7 | - | 529.5322 | 266.2486 | 244.2661 | 282.2431 |  |

**Supplementary Table 6*.***

| **ANTIBODY** | **CATALOG NUMBER** | **CLONE** |
| --- | --- | --- |
| Mouse anti-human CD45 BV421 | 563879 | HI30 |
| Mouse anti-human CD36 PE | 555455 | CB38 |
| Mouse anti-human CD11b BB515 | 564517 | ICRF44 |
| Mouse anti-human CD163 Alexa Fluor 647 | 568202 | MAC2-158 |
| Mouse anti-human CD69 PE | 555531 | FN50 |
| Rabbit anti-human ACADM | ab92461 | EPR3708 |
| Rabbit anti-human GAPDH | G9545 | polyclonal |
| Mouse anti-human CD163 | 760-4437 | MRQ-26 |
| Rabbit anti-E-chaderin 158Gd | 3158029D | 24E10 |
| Mouse anti-human CD20 161Dy | 3161029D | H1 |
| Rabbit anti-human CD4 156Gd | 3156033D | EPR6855 |
| Mouse anti-human CD8a 162Dy | 3162034D | C8/144B |
| Mouse anti-human CD68 159Tb | 3159035D | KP1 |
| Mouse anti-CD163 152Sm | 91H005152 | EDHu-1 |


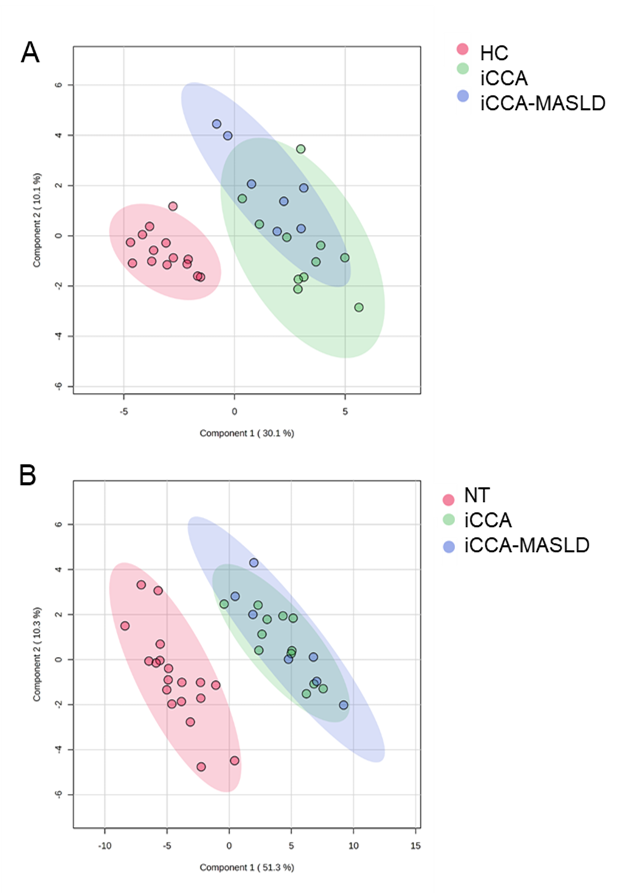


**Supplementary figure 1.** **Two-dimensional representation of the untargeted lipidomic profile of iCCA and MASLD-associated iCCA patients.** (A), [Partial Least Squares Discriminant Analysis](https://www.metabolon.com/bioinformatics/pls-da/) (PLSDA) of the serum lipidomic profile of HC (n=19), iCCA (n=12) and MASLD-associated iCCA (n=7) patients. (B), PLSDA of the tumor tissue lipidomic profile of NT, iCCA and MASLD-associated iCCA patients.

**
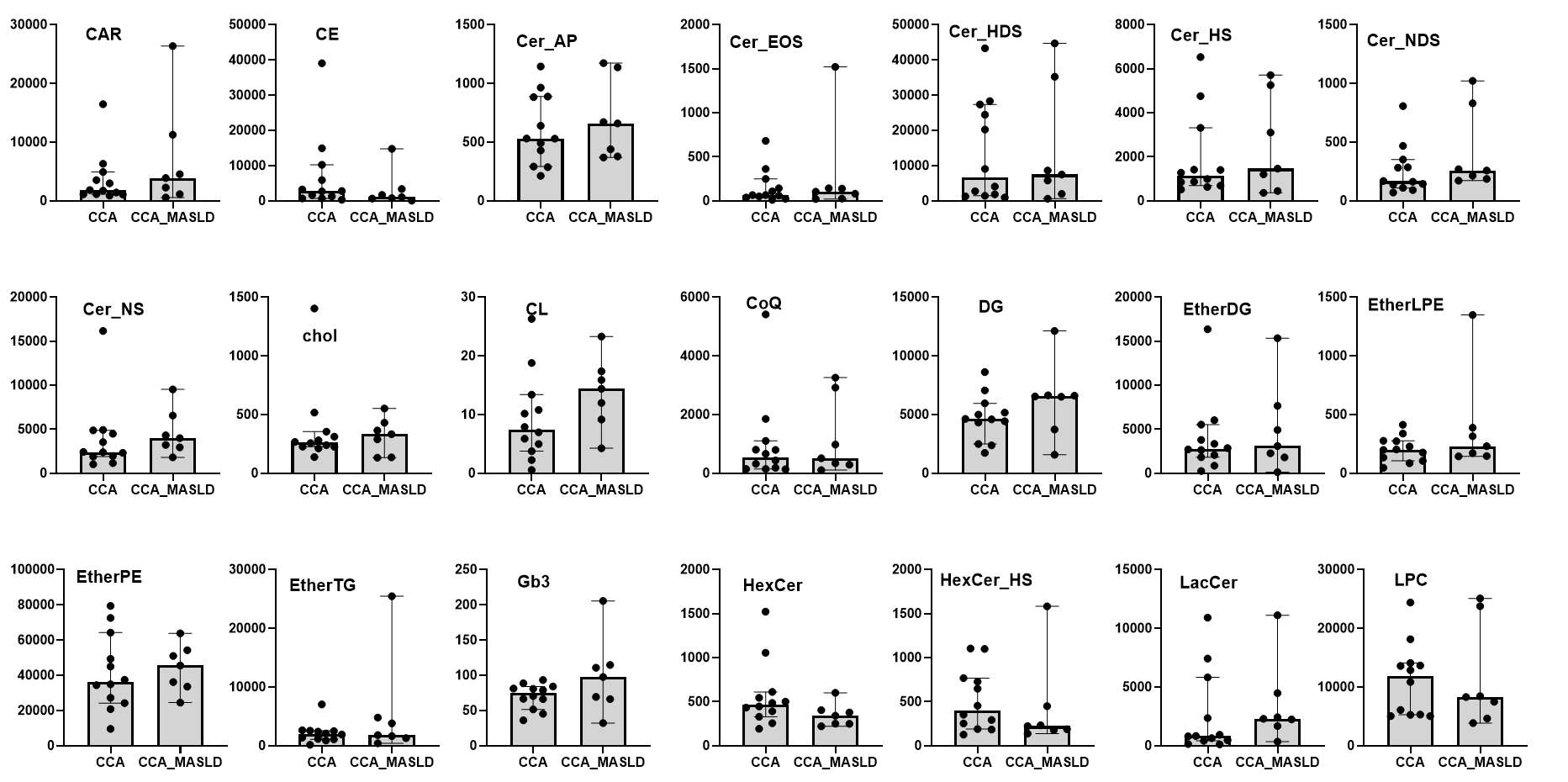
**

**
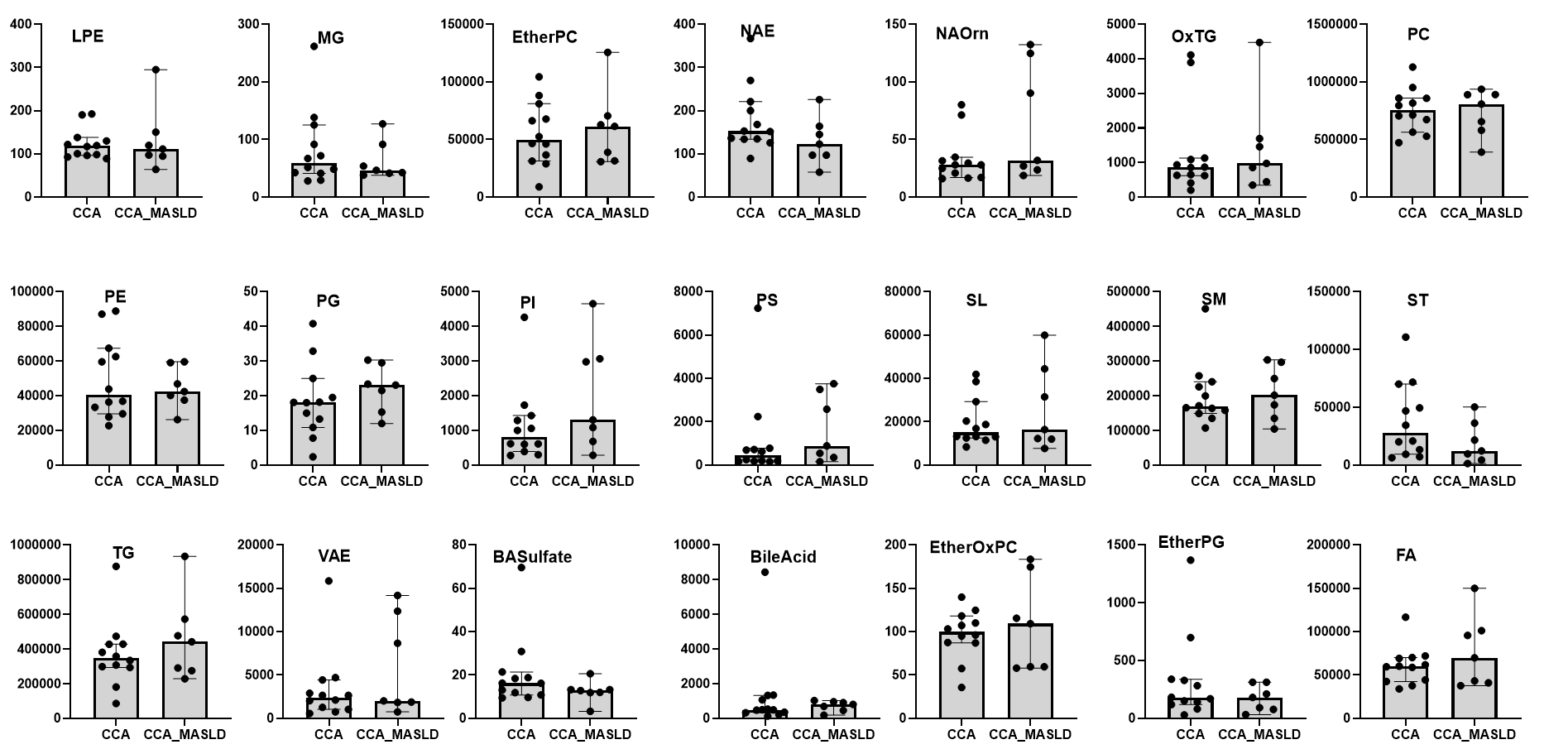
**

**
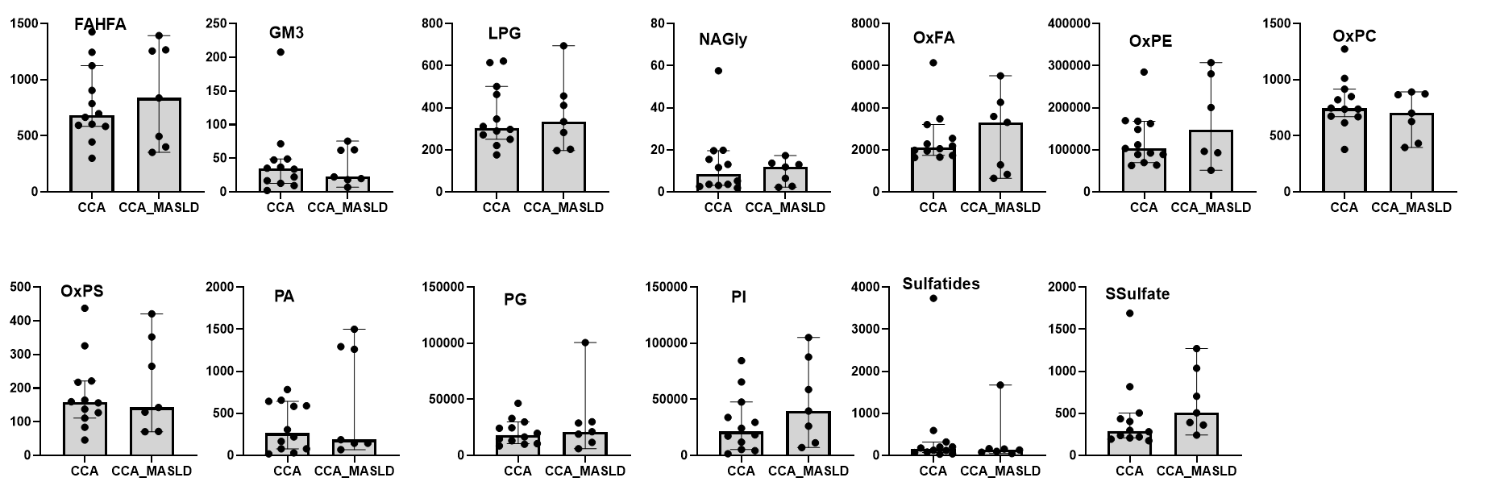
**

**Supplementary figure 2. Equal lipid profile of patients stratified by risk factors.** Analysis of 55 lipid classes in iCCA tissue (n=12) compared with MASLD-associated iCCA tissue (n=7) by high-resolution mass spectrometry. Shown are medians with 95% CI. The Mann-Whitney U test was used to compare data.


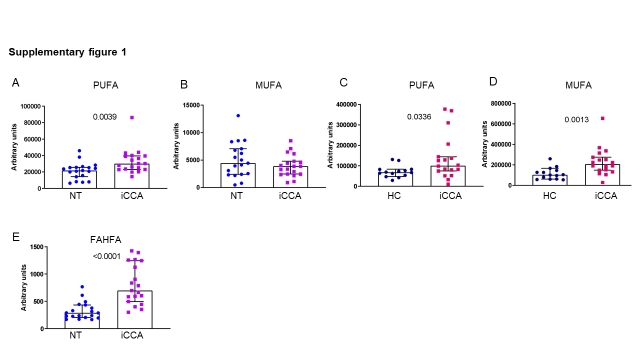


**Supplementary figure 3.** **PUFA, MUFA and FAHFA accumulate in iCCA tissues and sera.** Analysis of PUFA, MUFA and FAHFA in NT and matched iCCA tissues (**A**, **B,** **E;** n=11), and in HC and iCCA-derived sera (**C**, **D;** HC=23, iCCA=29). Shown are medians with 95% CI. The Wilcoxon test (**A**, **B, E**) or Mann-Whitney U (**C**, **D**) test were used to compare data.


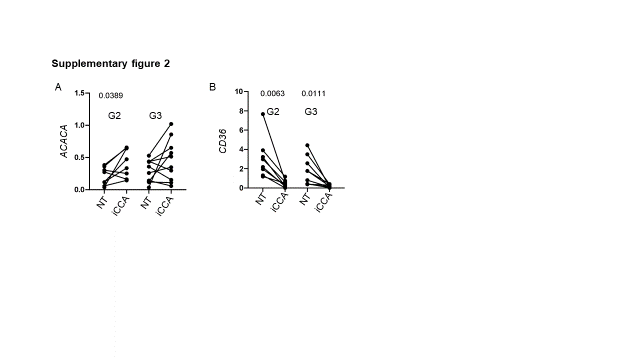
**Supplementary figure 4.** ***ACACA* and *CD36* are differently expressed in G2 and G3 iCCA compared with NT.** Gene expression analysis of *ACACA* (**A**) and *CD36* (**B**) in iCCA tissue stratified according to G stage compared to NT tissue (*ACACA*, iCCA-G2 n=8, iCCA-G3 n=8; CD36, iCCA-G2 n=8, iCCA-G3 n=9). The paired t test was used.


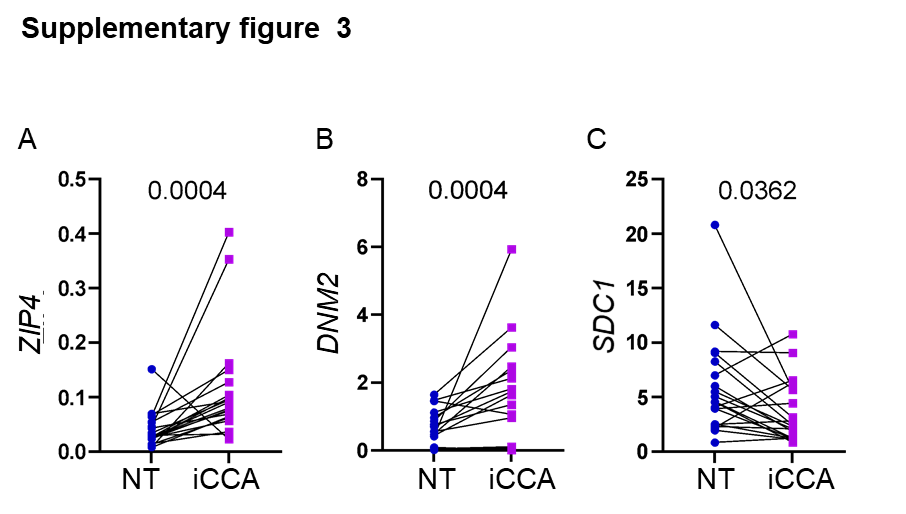


**Supplementary figure 5.** **Macropinocytosis-associated genes are differently expressed in iCCA tissue compared to NT.** Gene expression analysis of *ZIP4* (**A**), DNM2 (**B**) and *SDC1* (**C**) in iCCA tissues compared to NT (n=20). The Wilcoxon test was used to compare data.


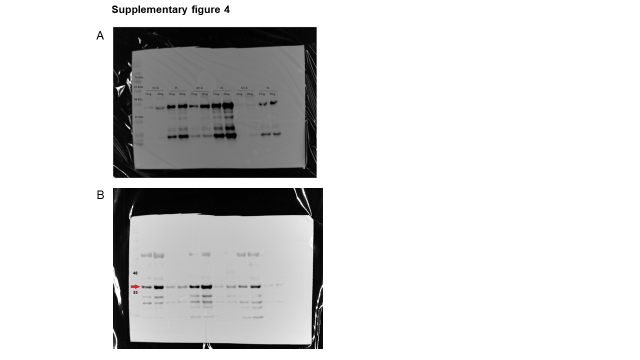


**Supplementary figure 6.** **ACADM is less expressed in iCCA tissues. A.** Original, uncropped blot of ACADM shown in figure 3. Lane 1 = standard ladders, lane 2, 6, 10 = 15 μg and 3, 7, 11 = 30 μg protein extract from three different iCCA biopsies. Lane 4, 8, 12 = 15 μg and 5, 9, 13 = 30 μg protein extract from the matched NT biopsies. **B.** Original, uncropped blot of the corresponding GAPDH for data normalization.


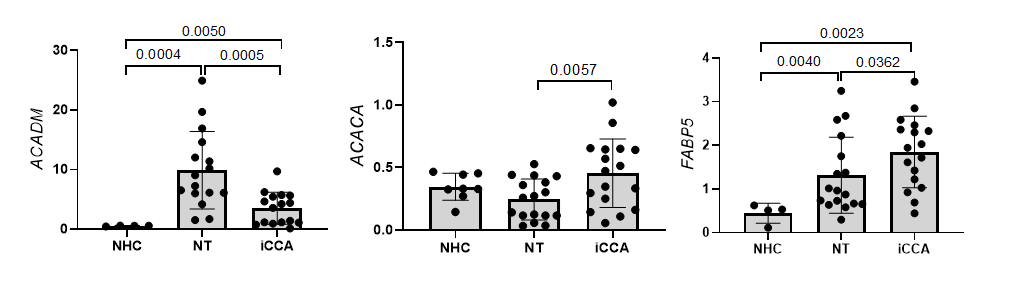


**Supplementary figure 7. Expression of genes related to lipid metabolism in iCCA tissue, normal human cholangiocytes (NHC) and NT.** Gene expression analysis of *ACADM* (**A**), ACACA (**B**) and *FABP5* (**C**) in NHC (n=4 for ACADM and FABP5 analysis; n=8 for ACACA analysis), iCCA tissues (n=16 for ACADM and FABP5 analysis; n=17 for ACACA analysis) and NT (n=16 for ACADM and FABP5 analysis; n=17 for ACACA analysis). The Mann-Whitney U test was used to compare NHC to NT and iCCA. The paired t test was used to compare NT to iCCA.


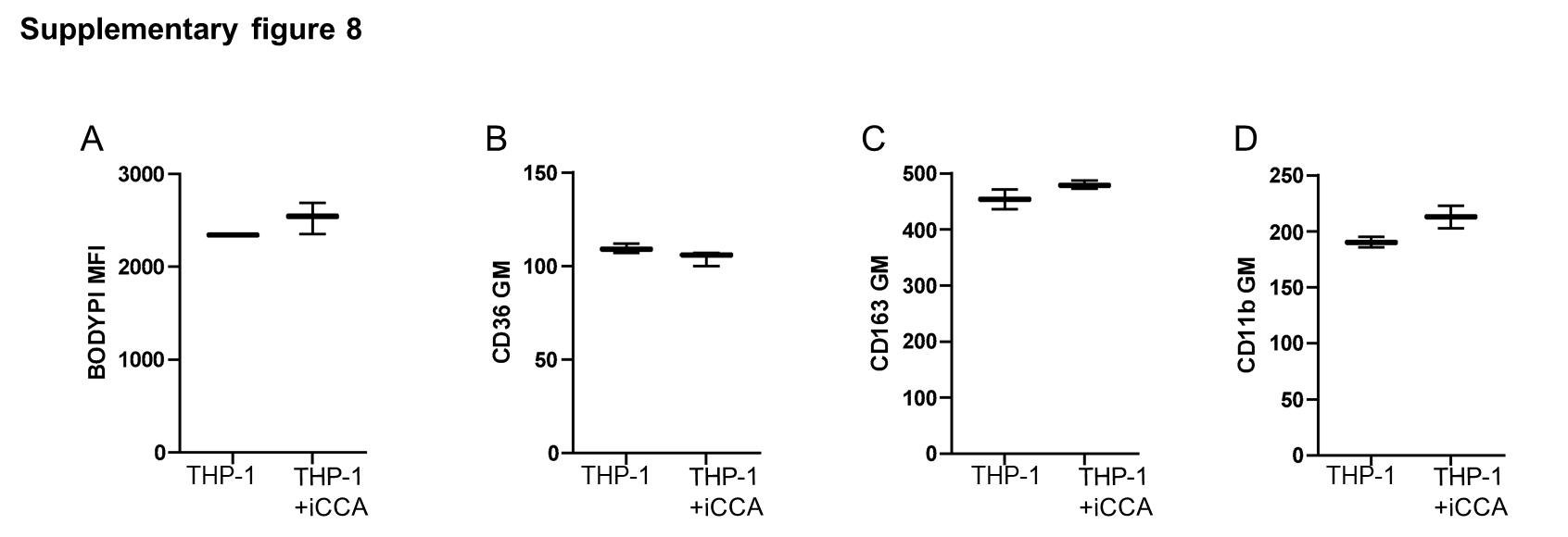


**Supplementary figure 8. Cell-cell contact is needed to induce alteration in THP-1 phenotype and lipid content.** Evaluation of BODIPY™505/515 (**A**), CD36 (**B**), CD163 (**C**) and CD11b (**D**) expression (MFI) on THP-1 cells after co-culture in the Transwell® system with primary iCCA cells.


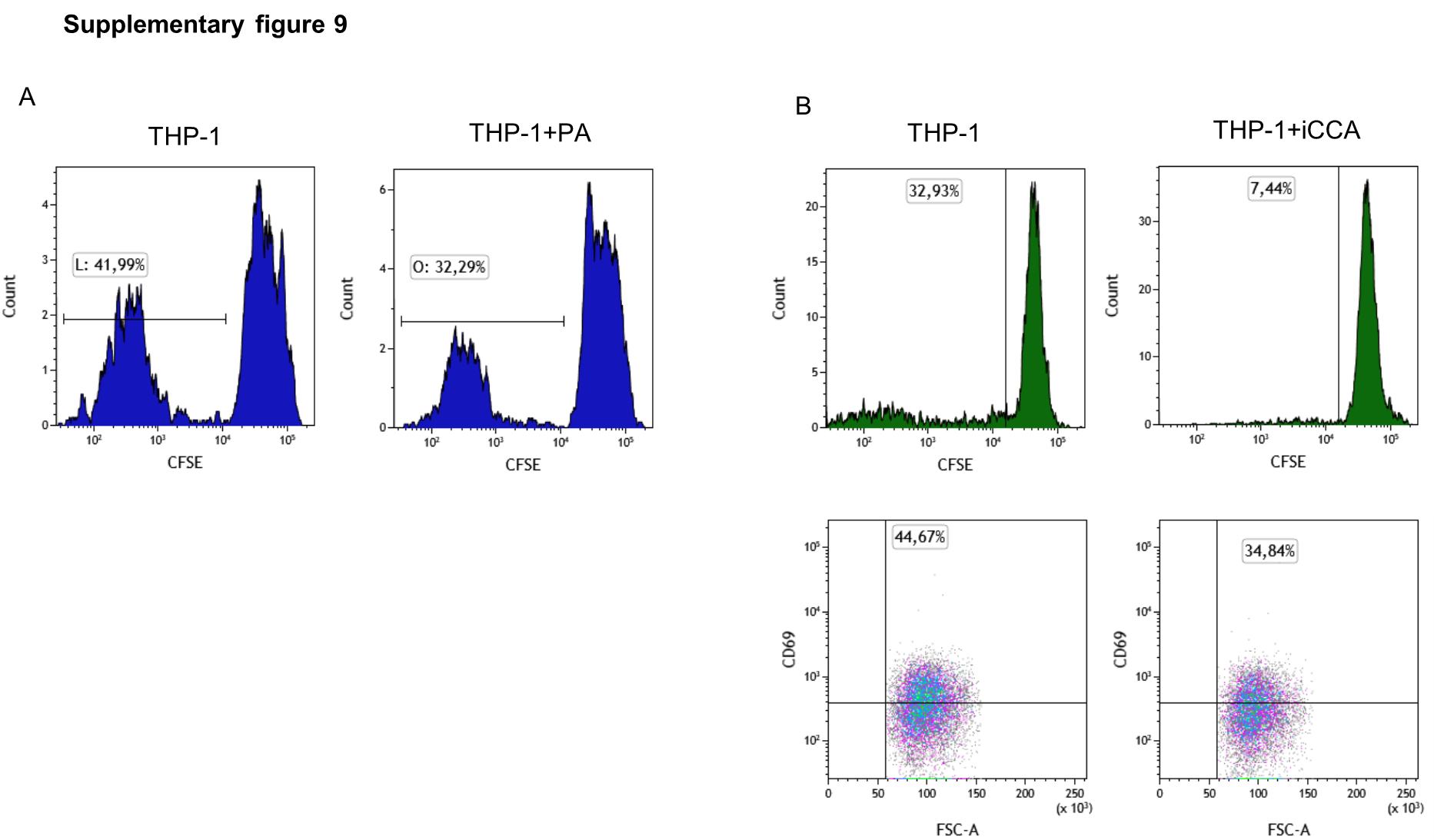


**Supplementary figure 9. THP-1 treated with PA or co-cultured with iCCA downregulated T cell proliferation. A:** T cell proliferation evaluated in CFSE-labeled PBMC stimulated with PHA (10 ug/ml) for 3 days in the presence of THP-1 or THP-1 pre-treated with PA. **B:** Upper panels represent the T cell proliferation in CFSE-labeled PBMC stimulated with anti-CD3/anti-CD28 stimuli for 3 days with THP-1 or THP-1 co-cultured with iCCA primary cells. Lower panels show activated (CD69+) T cells under identical experimental conditions.


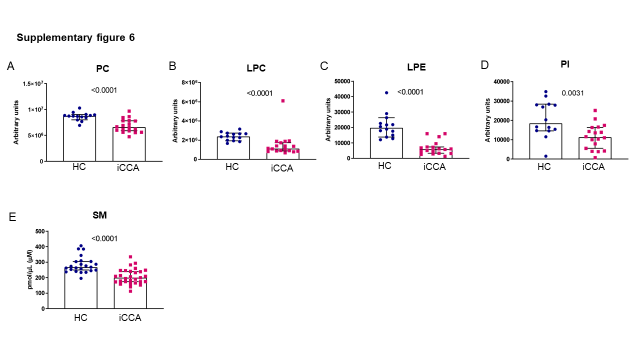


**Supplementary figure 10. Membrane forming lipids are downregulated in iCCA sera.** Analysis of different membrane forming lipid classes: PLs’ species (**A-D,** HC=14; iCCA=18) and SM (**E**, HC=23; iCCA=29) in HC and iCCA sera. Shown are medians with 95% CI. The Mann-Whitney U test was used to compare data.


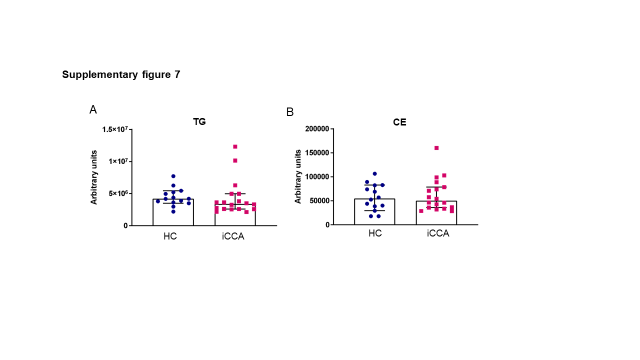


**Supplementary figure 11. Serum level of neutral lipids are unchanged in iCCA compared to HC.** Analysis of neutral lipids, TG and CE, in HC and iCCA sera (HC=14, iCCA=18). Shown are medians with 95% CI. The Mann-Whitney U test was used to compare data.


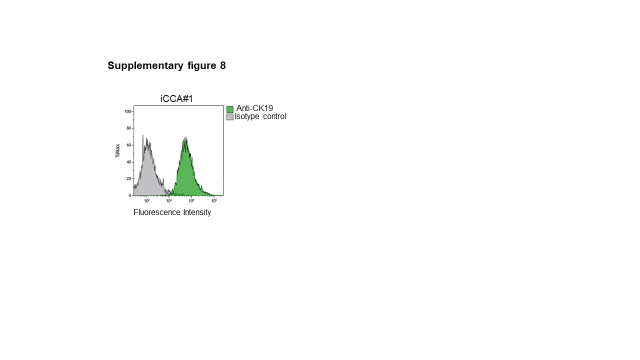


**Supplementary figure 12. CK19 expression on primary iCCA cells.** CK19 expression evaluated by flow cytometry on a patient-derived tumor cell culture.
